# P2Y_13_ receptors regulate microglial morphology, surveillance, and resting levels of interleukin 1β release

**DOI:** 10.1002/glia.23719

**Published:** 2019-09-14

**Authors:** Vasiliki Kyrargyri, Christian Madry, Ali Rifat, I. Lorena Arancibia‐Carcamo, Steffan P. Jones, Victor T. T. Chan, Yajing Xu, Bernard Robaye, David Attwell

**Affiliations:** ^1^ Department of Neuroscience, Physiology, & Pharmacology University College London London UK; ^2^ Department of Immunology, Laboratory of Molecular Genetics Hellenic Pasteur Institute Athens Greece; ^3^ Institute of Neurophysiology Charité – Universitätsmedizin Berlin Berlin Germany; ^4^ Faculté de Médecine Université Libre de Bruxelles Bruxelles Belgium

**Keywords:** ADP, ATP, P2Y_12_, P2Y_13_, purinergic signaling

## Abstract

Microglia sense their environment using an array of membrane receptors. While P2Y_12_ receptors are known to play a key role in targeting directed motility of microglial processes to sites of damage where ATP/ADP is released, little is known about the role of P2Y_13_, which transcriptome data suggest is the second most expressed neurotransmitter receptor in microglia. We show that, in patch‐clamp recordings in acute brain slices from mice lacking P2Y_13_ receptors, the THIK‐1 K^+^ current density evoked by ADP activating P2Y_12_ receptors was increased by ~50%. This increase suggested that the P2Y_12_‐dependent chemotaxis response should be potentiated; however, the time needed for P2Y_12_‐mediated convergence of microglial processes onto an ADP‐filled pipette or to a laser ablation was longer in the P2Y_13_ KO. Anatomical analysis showed that the density of microglia was unchanged, but that they were less ramified with a shorter process length in the P2Y_13_ KO. Thus, chemotactic processes had to grow further and so arrived later at the target, and brain surveillance was reduced by ~30% in the knock‐out. Blocking P2Y_12_ receptors in brain slices from P2Y_13_ KO mice did not affect surveillance, demonstrating that tonic activation of these high‐affinity receptors is not needed for surveillance. Strikingly, baseline interleukin‐1β release was increased fivefold while release evoked by LPS and ATP was not affected in the P2Y_13_ KO, and microglia in intact P2Y_13_ KO brains were not detectably activated. Thus, P2Y_13_ receptors play a role different from that of their close relative P2Y_12_ in regulating microglial morphology and function.

## Main points

- KO of P2Y_13_ receptors decreases microglial ramification and surveillance, slows chemotaxis to ADP and brain injury, and increases baseline release of interleukin 1β in brain slices.
- P2Y_13_ KO microglia generate larger ADP‐evoked THIK‐1 K^+^ currents.

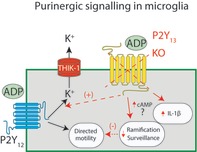

## 1 INTRODUCTION

Microglia are the resident immune cells of the central nervous system (CNS), with crucial roles in both tissue development and homeostatic maintenance. They express a wide array of membrane receptors, which allow them to sense their environment and respond to pathological stimuli. In the healthy brain, microglia are highly motile ramified cells that constantly extend and retract their processes to survey the CNS tissue (Davalos et al., 2005; Nimmerjahn, Kirchhoff, & Helmchen, 2005). We recently showed that this mode of microglial motility is regulated by the membrane potential generated by tonic activity of the two‐pore domain potassium channel THIK‐1 (Madry, Arancibia‐Cárcamo, et al., 2018; Madry, Kyrargyri, et al., 2018). Under pathological conditions, such as brain tissue damage, disease or infection, microglia become activated, upregulating surface receptors such as MHCII and A_2A_, while their morphology becomes less ramified, with fewer, shorter processes, and larger somata (Kreutzberg, 1996; Orr, Orr, Li, Gross, & Traynelis, 2009). Depending on the nature of the stimuli, microglia can also undergo a complete transformation into different functional and morphological, for example, amoeboid, states and secrete neurotoxic inflammatory, or neuroprotective anti‐inflammatory factors (Hanisch & Kettenmann, 2007).

ATP is a major neurotransmitter in the CNS, which is also released by injured cells and serves as a key stimulus for triggering microglial responses (Biber, Neumann, Inoue, & Boddeke, 2007; Burnstock, 2006; Zimmermann, 1994). Microglia detect ATP and its degradation product ADP through the purinergic receptor P2Y_12_ and respond by sending out their processes to surround regions of tissue damage: a process called directed motility (Haynes et al., 2006). P2Y_12_ is a G_i_‐protein‐coupled receptor (GPCRs), which inhibits adenylyl cyclase activity upon activation (Communi et al., 2001; von Kugelgen & Hoffmann, 2016). In addition to P2Y_12_ receptors, transcriptome data suggest that microglia express P2Y_13_ receptors at levels almost as high as for P2Y_12_ (Zhang et al., 2014). P2Y_12_ and P2Y_13_ receptors share a similar pharmacology and molecular structure (48% identical at the amino acid level; Perez‐Sen et al., 2017). However, although the function of P2Y_12_ receptors has extensively been studied, mainly because of its clinical relevance to platelet‐mediated thrombosis and the role of P2Y_12_ in directing microglial processes to damage sites, the significance of P2Y_13_ receptors for microglial function remains elusive.

Transcriptome data suggest that in the brain P2Y_13_ mRNA is expressed predominantly by microglia, much less by oligodendrocyte precursor cells and myelinating oligodendrocytes, and not by other brain cell types (Zhang et al., 2014). A recent study supports these data showing that microglia in the brain, and not neurons, astrocytes, or neural progenitor cells, express P2Y_13_ mRNA under basal conditions, using fluorescence in situ hybridization (Stefani et al., 2018). However, P2Y_13_ protein expression may be low (Haynes et al., 2006), possibly due to constitutive ubiquitination and proteasomal degradation (Pons et al., 2014). P2Y_13_ mRNA is upregulated in pathological conditions such as demyelination (Bedard, Tremblay, Chernomoretz, & Vallieres, 2007) and neuropathic pain (Kobayashi, Yamanaka, Yanamoto, Okubo, & Noguchi, 2012; Niu et al., 2017), suggesting a role of this receptor in neuroinflammation. However, its function in microglia, especially under physiological conditions, is still unclear, although it may evoke a rise of [Ca^2+^]_i_ in response to ADP (Zeng et al., 2014) and be involved in the release of proinflammatory cytokines (Liu et al., 2017). Increasing evidence also supports the notion that ADP may act through P2Y_13_ receptors to regulate the structural complexity of microglia (Matyash, Zabiegalov, Wendt, Matyash, & Kettenmann, 2017; Stefani et al., 2018) and contribute to the homeostatic control of adult hippocampal neurogenesis in situ (Stefani et al., 2018).

In this study, we expand this knowledge and describe the effect of genetically deleting P2Y_13_ receptors on microglial morphology and function in the brain. Knocking out P2Y_13_ results in less ramified microglial cells and a reduction in brain surveillance. It also increases the P2Y_12_‐mediated THIK‐1 current, although the directed motility (but not the speed of individual process movements) towards an ADP source or brain injury is slower in the P2Y_13_ KO mice, as the shorter processes need to grow further and thus take a longer time to reach their target. The membrane potential of P2Y_13_ KO microglia is similar to that of WT microglia, indicating that the deramified morphology is not due to voltage changes (Madry, Kyrargyri, et al., 2018), but has some aspects of a “primed‐like” microglial phenotype. This is further supported by higher baseline levels of interleukin 1β release in P2Y_13_ KO mice. However, in the intact brain of P2Y_13_ KO mice, no changes of classical microglial activation markers were detected. Together, these findings indicate that P2Y_13_‐deficient microglia exist neither in a typical nonactivated nor an activated state, but that they have adopted a partly deramified state which lies in between those two boundaries.

## 2 MATERIALS AND METHODS

### 2.1 Mice

Generation of the P2Y_13_ KO mouse strain and gross characterization were described previously (Fabre et al., 2010). In brief, they were generated through homologous recombination of a construct in which the first noncoding exon and 182 base pairs of the second exon of *p2ry13* were replaced with a neomycin resistance cassette to generate loss of P2Y_13_ expression. This construct was integrated into embryonic stem cell genomic DNA following electroporation. Blastocysts with this allele were generated and implanted into pseudopregnant females. Deletion of P2Y_13_ in this mouse line was confirmed via real‐time PCR of liver samples. (Confirmation of the deletion of microglial P2Y_13_ using antibody labeling or Western blots was not possible because neither of the two commercially available P2Y_13_ antibodies that we tested (Alomone APR017 and Abcam ab108444) nor the P2Y_13_ antibody, that was kindly provided by David Julius, labeled microglia (in brain slices or when isolated) or western blots of brain or spleen tissue specifically in the wild‐type mice; instead there was labeling of cells, and multiple western blot bands, in both WT and KO tissue (Figure S1). KO mice are healthy, fertile and show no behavioral abnormalities. For all experiments, mice with a C57BL/6‐background (backcrossed at least 12 times) were used, and WT and P2Y_13_ KO mice of either sex were age‐matched and usually littermates. For microglial imaging in real‐time, for some experiments, the P2Y_13_ KO mice were bred with transgenic mice expressing eGFP under control of the Iba1 promoter (Hirasawa et al., 2005) in which microglia are labeled by eGFP. Preweaning animals were housed with their mother and sometimes the father as well; weaned animals were housed in groups of 2–5. Housing was in individually ventilated cages. Animal procedures were carried out in accordance with the guidelines of the UK Animals (Scientific Procedures) Act 1986 and subsequent amendments, and a local ethical review board gave approval. Ages are stated in the figure legends for each experiment.

### 2.2 Brain slice preparation

Acute hippocampal slices (300 μm thick) from young animals (1–2 months old) were prepared using an ice‐cold standard slicing solution (Bischofberger, Engel, Li, Geiger, & Jonas, 2006), containing (mM) 124 NaCl, 26 NaHCO_3_, 1 NaH_2_PO_4_, 2.5 KCl, 2 MgCl_2_, 2 CaCl_2_, 10 glucose, bubbled with 95% O_2_/5% CO_2_, pH 7.4, as well as 1 mM Na‐kynurenate to block glutamate receptors. Hippocampal slices from older animals (>2 months old) were prepared using two different solutions (Nortley et al., 2019; Ting, Daigle, Chen, & Feng, 2014), a protective NMDG slicing solution containing (mM) 93 N‐methyl‐d‐glucamine (NMDG), 93 HCl, 20 HEPES, 30 NaHCO_3_, 2.5 KCl, 0.5 CaCl_2_, 10 MgCl_2_, 1.2 NaH_2_PO_4_, 25 glucose, 1 kynurenic acid, 5 Na‐ascorbate, 3 Na‐pyruvate, pH adjusted to 7.4 with HCL, bubbled with 95%O_2_/5%CO_2_ and cooled at <4°C and a protective recovery solution containing (mM) 92 NaCl, 20 HEPES, 30 NaHCO_3_, 2.5 KCl, 2 CaCl_2_, 1 MgCl_2_, 1.2 NaH_2_PO_4_, 25 glucose, 1 kynurenic acid, 5 Na‐ascorbate, 3 Na‐pyruvate, pH set to 7.4 with NaOH, bubbled with 95%O_2_/5%CO_2_. Immediately after slicing, slices were moved to warmed slicing solution (33–35°C) for 15 min before moving them to the recovery solution at room temperature where they were stored until the experiment. The kynurenic acid was added to the slicing solutions to block glutamate receptors and prevent excitotoxic damage to neurons during the slicing but it was removed from the experimental solution as described below. At the age of 2–4 months old, P2Y_13_ mRNA expression is relatively high (Crain, Nikodemova, & Watters, 2009). Brain slicing does not activate microglia for at least 4 hr, as judged by cell morphology, motility and interleukin 1β release (Gyoneva & Traynelis, 2013; Kurpius, Wilson, Fuller, Hoffman, & Dailey, 2006), and allows pharmacological investigation of mechanisms in a manner that is not possible using in vivo experiments. For experiments not employing Iba1‐GFP mice (as stated in the main text), slices were incubated for 30 min in darkness at room temperature (22–24°C) in oxygenated HEPES‐buffered external solution (see below) containing 25 μg/ml Alexa 594 conjugated isolectin B_4_ (ThermoFisher) for live imaging experiments or 25 μg/ml Alexa 568 conjugated isolectin B_4_ for electrophysiology experiments (Grinberg, Milton, & Kraig, 2011; Kurpius et al., 2006), before being used in experiments. Isolectin B_4_ labeling does not activate microglia (Grinberg et al., 2011) and its use avoids functional changes which might occur in transgenically labeled microglia.

### 2.3 Solutions and electrophysiology

Slices were superfused with HEPES‐buffered solution, at 34–36°C for all experiments involving imaging and at room temperature (22–24°C) for electrophysiological experiments, containing (mM) 140 NaCl, 2.5 KCl, 10 HEPES, 1 NaH_2_PO_4_, 2 CaCl_2_, 1 MgCl_2_, 10 glucose, pH set to 7.4 with NaOH, bubbled with 100% O_2_. Cells were whole‐cell clamped with electrodes containing KCl based solution, comprising (mM) 125 KCl, 4 NaCl, 1 CaCl_2_, 10 HEPES, 10 EGTA, 4 MgATP, 0.5 Na_2_GTP, pH set to 7.1 with KOH. The final osmolarity was 285 ± 5 mOsmol/kg. Microglia were identified by their fluorescent label and ramified morphology, and whole‐cell clamped at a depth of ~50–100 μm in the slice using borosilicate pipettes with a tip resistance of ~4–5 MΩ, giving a series resistance of <20 MΩ. Electrode junction potentials were compensated. *I*–*V* relations were from responses to 200 msec voltage steps ranging from −124 mV to +56 mV in 30 mV increments. Voltage‐ and current‐clamp recordings were performed using a MultiClamp 700B amplifier (Molecular Devices). Currents were filtered at 1 kHz, digitized (10 kHz) and analyzed off‐line using pClamp10 software.

### 2.4 Two‐photon imaging and evoked directed motility

Microglia in hippocampal slices were imaged at 34–36°C, at a depth of ~50–100 μm in the slice (to avoid studying superficial microglia that had started to become activated by the slicing procedure) using a Zeiss LSM 710 or 780 microscope (with a 20X lens, NA 1.0) and a Spectraphysics Mai Tai DeepSee eHP Ti:sapphire infrared laser. For imaging of microglia labeled with isolectin B_4_‐Alexa 594, the laser was tuned to a wavelength of 800 nm, while for imaging cells labeled with eGFP a wavelength of 920 nm was used with a pixel dwell time of 1 μs. Generally the laser was adjusted to 1.8% of its maximum power at 800 nm or 6–8% at 920 nm, corresponding to ~5 mW and ~12 mW, respectively, at the preparation, that is, well within the intensities used by others (Hines, Hines, Mulligan, & Macvicar, 2009; Pfeiffer, Avignone, & Nagerl, 2016; Wake, Moorhouse, Jinno, Kohsaka, & Nabekura, 2009).

Ablation of a small volume of tissue (laser damage) was performed by illuminating a ~10 μm radius spot with the laser intensity increased 30‐fold and the pixel dwell time increased to 100 μs. For imaging of directed process motility in response to a pipette filled with 1 mM ADP or to a laser ablation, stacks of 21–31 slices imaged at 2 μm depth intervals were acquired every 30 s (the image analysis was performed to maximum intensity of stacks having a total *z*‐dimension of 12 μm as described below). For imaging of microglial surveillance, stacks of 21–31 slices imaged at 2 μm depth intervals were acquired every 60 s. Images were typically 512 by 512 pixels and covered a square field of view 200–250 μm wide.

### 2.5 Image analysis

Analysis of two‐photon images was performed using custom‐written ImageJ (NIH) and MATLAB scripts, available at https://github.com/AttwellLab/Microglia. For analysis and quantification of microglial surveillance, each slice of every stack was filtered with a median filter after subtraction of smooth continuous background with the ImageJ “subtract background” plugin with a ball size of 30 pixels. The 4D stacks were then registered first for lateral drift, then rotated 90° on their side, registered for z‐drift and rotated back to their original orientation. We then performed a maximum intensity projection. Cells of interest were individually selected by manually drawing a region of interest (ROI) around an area including all their process extensions over the whole duration of the resulting 2D movie, and erasing data around that ROI. These 2D movies of individual cells were then manually binarized and saved as independent files. The binarization was performed on high resolution and preprocessed images with the experimenter blinded to the genotype. The choice of the threshold value was based on the intensity and morphology of the cells. As a general binarization criterion, the threshold value was set to a value ensuring that all the microglial processes, even the thinner ones, were visible in all the different time frames.

To quantify surveillance, for each movie, starting with the second frame, we subtracted from each binarized frame *F*_t_ the preceding frame *F*_t − 1_ and created two binarized movies, *PE* consisting of only the pixels containing process extensions (*F*_t_ − *F*_t − 1_ > 0) and *PR* consisting of only the pixels containing process retractions (*F*_t_ − *F*_t − 1_ < 0). In both *PE* and *PR*, all other pixels are set to 0. The surveillance index *B* is defined as the sum over all nonzero pixels in *PE* + *PR*, that is:

$$B=\sum_{\text{pixels}}\left(\mathrm{PE}+\mathrm{PR}\right)$$

where ∑_pixels_ denotes a sum taken over all nonzero pixels, and in most graphs is displayed normalized to its average over the first 20 min of the experiment (<∑_pixels_ [*PE* + *PR*] > _control_ where < >_control_ denotes a temporal average taken over the 20‐min control period of the experiment), or normalized to the value for WT mice. The surveillance index provides a measure of the brain volume that is surveyed by a microglial cell in a given time. It is affected both by the rate at which processes elongate and shorten, and by the overall number of the microglial processes and their length.

To quantify ramification, the resulting movies were processed using MATLAB. In each movie frame, the MATLAB functions bwarea (http://uk.mathworks.com/help/images/ref/bwarea.html) and bwperim (http://uk.mathworks.com/help/images/ref/bwperim.html) with 8‐connected neighborhood were used to quantify, respectively, the area and the perimeter of the cell. The ramification index *R* is defined as the ratio of the perimeter to the area, normalized by that same ratio calculated for a circle of the same area. Specifically:

$$R=\left(\text{perimeter}/\text{area}\right)/\left[2.{\left(\pi /\text{area}\right)}^{1/2}\right].$$

Thus, *R* = 1 if the cell is a perfect circle. The more ramified the cell, the larger *R* is.

To quantify the area‐normalized motility index, a measure of how well the cell surveys the brain given its size, we normalized the surveillance index of each individual cell to its mean area. The area of each cell was measured in maximum intensity projections for every time frame and then averaged over all time frames to get the mean area (in pixels) per cell. We then averaged the ratios (surveillance index/mean area) of all cells for the WT and all cells for the KO mice and reported these numbers.

For analysis and quantification of microglial directed process motility in response to a laser ablation to simulate brain injury, or to a pipette filled with 1 mM ADP, each slice of every stack was first filtered with a median filter (which replaces each pixel value with the median value of the 3 pixel × 3 pixel array centered on that pixel). We then performed a maximum intensity projection of stacks having a total *z*‐dimension of 12 μm (with the pipette tip or the laser ablation in the middle), registered (Thevenaz, Ruttimann, & Unser, 1998), and binarized the resulting two‐dimensional movies (setting the threshold for binarization manually). The resulting movies were processed using a MATLAB script inspired by the algorithm described by Gyoneva et al. (2014). Briefly, after the user manually clicks on the final target of chemotactic processes (the tip of the glass pipette containing ADP), the algorithm divides the surrounding area into concentric circles with radii at 2 μm intervals, and then segments these circles into 32 radial sectors, thus creating 32 patches between every two consecutive concentric circles. Then, for each frame, starting from the center, the algorithm searches in every radial sector for the first patch containing >10 positive pixels (labeled microglia). The outputs of the algorithm are, for each frame, (a) the surface area contained within the converging microglial process front (Figure 4a,c) and (b) the distance to the microglial process front in each sector (Figure 4b,d). To estimate the speed of individual process movements (for Figure 4g), in each sector the rate of approach (in μm/30 s) of the microglial processes was calculated for 30 s time bins, averaged over all sectors to obtain an average for that cell and time bin, and then averaged over all time bins during the convergence process (until no further convergence was observed) for that cell, and then averaged over all cells.

### 2.6 Microglia immunostaining and Sholl analysis

Microglial morphology was assessed by Sholl analysis (Sholl, 1953). To compare changes in microglial morphology produced by deletion of P2Y_13_ expression, three KO and three control wild‐type (WT) mice were killed by sodium‐pentobarbital overdose and fixed by transcardial perfusion with 4% paraformaldehyde (PFA). Brains were then removed and further fixed for 24 hr in 4% PFA. Horizontal sections (75 μm, “magic cut”; Bischofberger et al., 2006) were cut on a vibratome (Leica). Tissue sections were blocked and permeabilized in 10% goat serum and 0.5% Triton X‐100 in PBS for 4 hr at room temperature. Slices were incubated at 4°C overnight in rabbit anti‐Iba1 (Synaptic Systems 234003) antibody prepared in blocking solution. After washing three times for 20 min in PBS, slices were incubated overnight in secondary antibody (goat anti‐rabbit Alexa Fluor 488). Slices were then washed in PBS, incubated in DAPI (Invitrogen) and mounted on glass slides using DAKO fluorescence mounting medium. All images were acquired using a 63× oil immersion objective (NA 1.4) on a Zeiss LSM700 confocal microscope. All analysis was carried out with the experimenter blinded to the genotype. Cell reconstructions were carried out using 3D automatic cell tracing in Vaa3D software (http://www.vaa3d.org), which is available online and uses the APP2 (all‐path‐pruning 2.0) algorithm to re‐construct ramified cells in 3D (Xiao & Peng, 2013). In brief, the algorithm uses the gray‐weighted image distance transform (https://uk.mathworks.com/help/images/ref/graydist.html) to assess the area occupied by the cell. This can be done either automatically or manually. It then allows the experimenter to manually perform a background thresholding method and applies the distance transform algorithm to specify the cell body as the main seed node. It subsequently reconstructs a “tree” where the seed node is the cell body, the branch nodes are the microglial branches that connect different processes (segments) and the leaf nodes are the terminals. It then applies a length‐based hierarchical pruning method to code the final reconstruction of the cell. Finally, the tracing method produces a file with all the hierarchical information stored and morphological parameters are then extracted using custom code written in MATLAB. The code used is available at https://github.com/AttwellLab/Microglia.

### 2.7 P2Y_13_ immunohistochemistry

Fluorescent immunohistochemistry was performed on vibratome hippocampal slices (70 μm) from perfusion‐fixed WT and P2Y_13_ KO mice (Figure S1a). Slices were initially incubated in antigen retrieval solution containing 10 mM Na citrate and 0.05% Tween20, pH 6 at 90°C for 20 min. The slices were then washed three times in PBS, blocked and permeabilized in 10% horse serum and 0.5% Triton X‐100 in PBS for 2 hr at room temperature and incubated in primary antibodies to rabbit P2Y_13_ (1:20, APR017, Alomone Labs) and to chicken Iba1 (1:500, Novus biologicals) overnight at 4°C. The slices were then washed three times in PBS for 20 min and incubated in secondary antibodies (1:1,000, donkey anti‐rabbit Alexa‐Fluor 568 and donkey anti‐chicken Alexa Fluor 488). Slices were then washed in PBS, incubated in DAPI (Invitrogen) and mounted on glass slides using DAKO fluorescence mounting medium.

DAB Immunohistochemistry was performed on brain paraffin sections (5 μm) from WT and P2Y_13_ KO mice to detect P2Y_13_ protein expression in situ (Figure S1d–f). The sections were initially de‐paraffinized in xylene and re‐hydrated in a descending ethanol series (100, 96, 70, and 50%) and dH_2_O. Endogenous peroxidase activity was blocked by incubation of the slides in methanol/0.2% H_2_O_2_ for 30 min. Antigen retrieval was performed in a food steamer with 10 mM Tris/1 mM EDTA buffer (pH 8.5) for 20 min. The slices were then washed in PBS, blocked in 10% FBS in PBS (blocking buffer) for 1 hr at room temperature and incubated in primary rabbit anti‐P2Y_13_ (that was kindly provided by Prof Julius lab, UCSF) overnight at 4°C. The sections were then washed in PBS and incubated with biotinylated anti‐rabbit secondary antibody (1:1,000 in blocking buffer, Vector Laboratories) for 1 hr, at room temperature. After washing in PBS, an avidin–biotin complex was used for detection of the biotinylated antibodies and immune complexes were visualized by brief incubation of the slices in 3,3′‐diaminobenzidine tetrachloride (DAB; both from Vector Laboratories). Nuclei counterstaining was performed using Mayers Hematoxylin (Abcam).

### 2.8 Microglia isolation from adult mouse brain and immunocytochemistry

Microglia isolation from adult mice was performed following the protocol described in Lee and Tansey (2013). In brief, three adult WT and three adult KO mice were perfused with ice‐cold sterile PBS under deep anesthesia (sodium pentobarbital). Mice were then killed and brains removed and finely minced with a blade on ice, followed by incubation of the tissues in dissociation medium containing DNase I (20 U/ml, Invitrogen), Dispase II (1.2 U/ml, Roche), and Papain (1 mg/ml, Sigma‐Aldrich) for 30 min at 37°C. Tissues were subsequently mechanically dissociated (with the use of Pasteur pipettes) and filtered into single‐cell suspensions before being centrifuged at 250 g for 4 min. The cell pellets were then re‐suspended in 37% isotonic Percoll (4 ml per brain) and loaded into 15 ml conical tubes in between 30 and 70% layers of Percoll. The Percoll gradients were then centrifuged at 300*g* for 40 min at 18°C. The 70–37% interfaces, that included microglia, were collected, diluted in HBSS (1×, Sigma Aldrich) and centrifuged at 500*g* for 7 min at 4°C. The pellets were re‐suspended in HBSS, washed three times and then seeded on poly‐l‐lysine precoated coverslips in 24‐well plates and used for immunocytochemistry.

For double immunofluorescence, WT and P2Y_13_ KO cells were fixed with 4% PFA for 20 min at 4°C, washed three times in PBS, blocked and permeabilized in 10% horse serum and 0.5% Triton X‐100 in PBS for 4 hr at room temperature and incubated in primary antibodies rabbit P2Y_13_ (1:20, APR017, Alomone Labs) and goat Iba1 (1:50, Novus Biologicals) overnight at 4°C. The cells were then washed 3 times in PBS and incubated in secondary antibodies (donkey anti‐rabbit Alexa Fluor 568 and donkey anti‐goat Alexa Fluor 488) for 1 hr at room temperature. Cells were then washed in PBS, incubated in DAPI (Invitrogen) and mounted on glass slides using DAKO fluorescence mounting medium. All images were acquired using a 63× oil immersion objective (NA 1.4) on a Zeiss LSM700 confocal microscope.

### 2.9 ELISA measurements of interleukin‐1β and TNFα release

As previously described (Charolidi, Schilling, & Eder, 2015; Madry, Kyrargyri, et al., 2018), hippocampal slices (300 μm thick) were prepared in ice‐cold HEPES‐buffered medium (MEM bubbled with O_2_, pH 7.4, 42360‐032, Gibco) under sterile conditions. To induce inflammasome activation and IL‐1β release, slices were exposed to inflammatory‐like stimuli (Bernardino et al., 2008). Each slice was placed on a Millicell cell culture insert (12 μm pore size, PIXP01250, Merck Millipore) and transferred into 24‐well plates containing 800 μl serum‐free medium (DMEM, pH 7.4, 41,965–039, Gibco) with or without lipopolysaccharide (LPS; 50 μg/ml, *Escherichia coli* 055:B5, L2880, Sigma‐Aldrich) in a cell culture incubator at 37°C. After 30 min, the medium above the slices was removed and slices were kept for 6 hr in 350 μl DMEM with or without LPS, for the last 3 hr of which 1 mM ATP was added (or not), as indicated. The concentrations of LPS used were chosen to evoke reliably detectable release of IL‐1β, and are similar with those that have been described in the literature to induce inflammatory response for mice (Hines, Choi, Hines, Phillips, & MacVicar, 2013). The amounts of IL‐1β and TNFα released into the medium were measured by ELISA, using mouse Quantikine IL‐1 beta/IL‐1F2 (R&D Systems MLB00C) and mouse TNFα Quantikine (MTA00B) kits, respectively. Data were from at least three mice per experiment, from each of which two brain slices were used per experimental condition. Immediately after collecting the media, photographs of slices were taken and the slice surface area was determined using Image J. To compare data between brain slices in different conditions, the amounts of IL‐1β and TNFα released into the medium were normalized to the slice surface area. The data were then further normalized to the mean of the control values obtained in WT mice.

### 2.10 RNA isolation and quantitative RT‐PCR

Total RNA was extracted from whole‐brain tissues using TRIzol (Invitrogen, Paisley, UK) according to the manufacturer's instructions. DNAse‐treated RNA samples were analyzed by quantitative RT‐PCR using QuantiFast™ SYBRVR green RT‐PCR kit (Qiagen Inc.) according to the manufacturer's instructions. All reactions were performed using a LightCycler (Roche, Mannheim, Germany). At the end of each PCR run, melting curve analysis was performed to verify the integrity and homogeneity of PCR products. Gene expression levels were calculated using standard curves for each gene, which were created by plotting threshold cycle (CT) values versus the logarithm of serial‐diluted RNA concentrations. A least‐square method was used for the determination of *A* and *B* values in the equation CT = A*Log (C_RNA_) + *B*. The coefficient of determination (*R* ^2^) was greater than 0.99. Values were normalized using the respective values for the housekeeping gene, Gapdh. All results were analyzed using the LightCycler software version 3.5 (Roche, Mannheim, Germany, RRID: rid_000088). QuantiTect Primer Assays were used for Il6 (Mm_Il6_1_SG), Il1b (Mm_Il1b_2_SG), tlr2 (Mm_Tlr2_1_SG), Csf1 (Mm_Csf1_2_SG), Lgals3 (Mm_Lgals3_1_SG), P2ry12 (Mm_P2ry12_3_SG), Nos1 (Mm_Nos1_2_SG), and Gapdh (Mm_Gapdh_3_SG), all QuantiTect Primer Assays from Qiagen.

### 2.11 Western blot

Twenty micrograms of total protein extract from whole brain and spleen tissues were resolved on NuPAGE 4–12% Novex Bis‐Tris Gels (Invitrogen, NP0321) and transferred onto nitrocellulose membranes. Blots were probed with antibodies to P2Y_12_ (kindly provided by Dr. David Julius, UCSF) and to P2Y_13_ (Abcam, ab108444) followed by horseradish peroxidase‐conjugated goat anti‐rabbit IgG secondary antibody (1:2,000, Thermo Fisher Scientific, 65‐6120). Antibody binding was detected using the Luminata Crescendo Western HRP substrate (Sigma, WBLUR0500). To normalize for protein content, membranes were stripped and re‐probed with anti‐actin antibody (1:5,000, Sigma, A2066). Quantification of the protein expression level was done with ImageJ software using the built‐in Gel Analysis protocol.

### 2.12 Statistics

Data are presented as mean ± SEM. *p* values are from two‐tailed Student's *t* tests (for normally distributed data) or Mann–Whitney U tests (for non‐normally distributed data). Normality of data was checked using the Kolmogorov–Smirnov or Shapiro–Wilk test and equality of variance confirmed using the F‐test. Sholl analysis distributions were compared with a Kolmogorov–Smirnov test. For multiple comparisons, *p* values are corrected using a procedure equivalent to the Holm–Bonferroni method (for *N* comparisons, the most significant *p* value is multiplied by *N*, the second most significant by *N* − 1, the third most significant by *N* − 2, etc.; corrected *p* values are significant if they are <0.05).

## 3 RESULTS

### 3.1 P2Y_13_ receptor knock out decreases microglial ramification and surveillance

**Figure 1:**
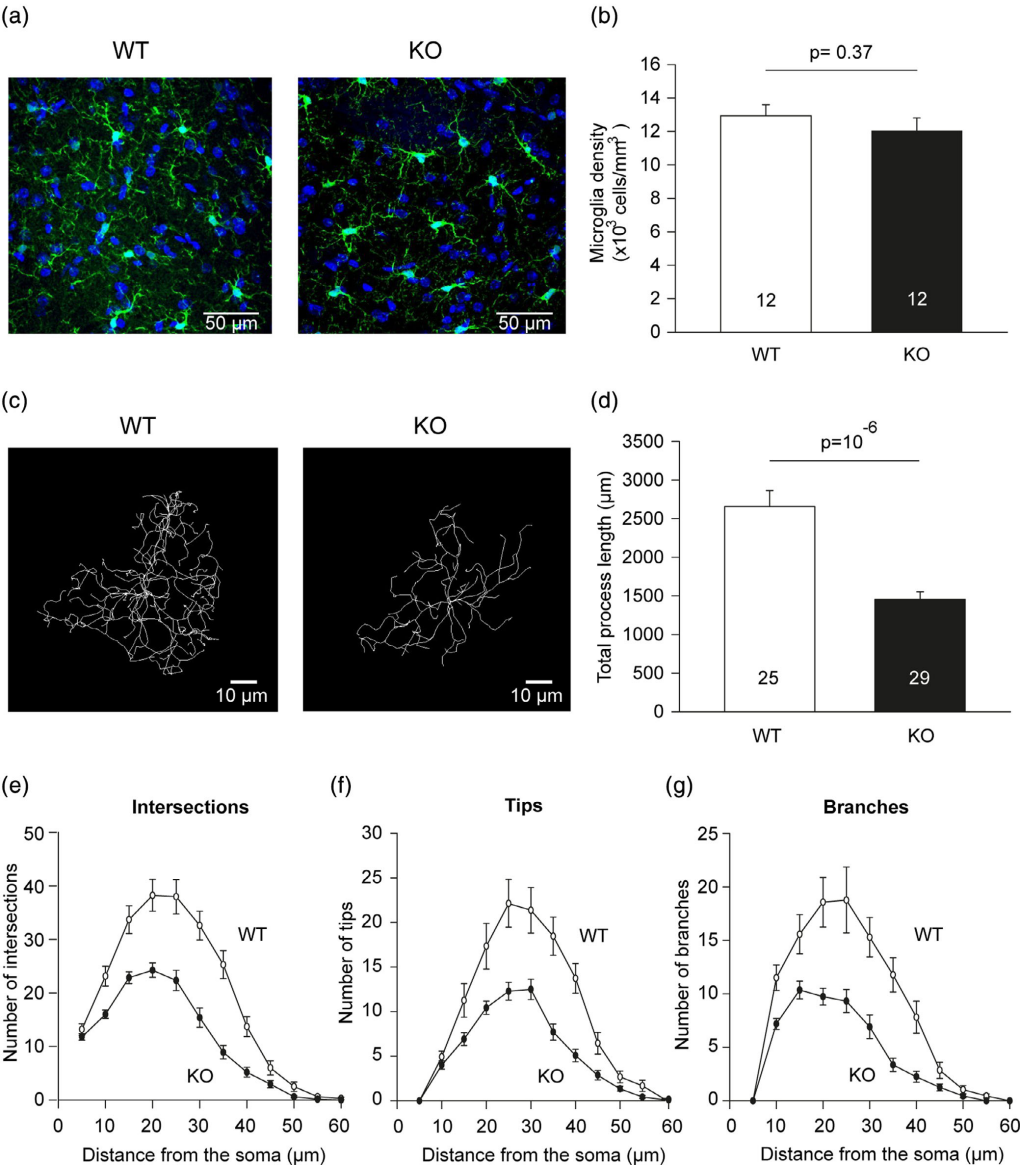
Effect of P2Y_13_ receptor knock‐out on microglial morphology and density in hippocampal slices. (a) Specimen images of perfusion‐fixed P90 wild‐type (WT) and P2Y_13_ knock‐out (KO) hippocampal slices labeled for Iba1 show that microglial density appears unchanged in the KO. (b) Microglial density in the strata radiatum and lacunosum/moleculare of areas CA1–CA3 of 12 WT and 12 KO hippocampal slices (from 4 WT and 4 KO P90 animals). Numbers of slices are on bars. (c–g) Ramification analysis of microglia from perfusion‐fixed WT (25 cells at P59–P135) and P2Y_13_ KO (29 cells at P53–P127) mice, showing (c) representative 3D‐reconstructed P59 WT and P53 KO microglia, and (d–g) Sholl analysis derived: (d) total process length; (e) number of process intersections with shells at distances (in 5 μm increments) from the soma; (f) number of process terminal points (tips); and (g) number of branches [Color figure can be viewed at http://wileyonlinelibrary.com]

To test for an effect of knocking out P2Y_13_ receptors on microglial morphology, we perfusion fixed adult wild‐type (WT) and P2Y_13_ KO mice, immuno‐labeled microglia for Iba‐1 in hippocampal slices, and quantified their process ramification by performing a three‐dimensional Sholl analysis. Individual cells were analyzed in high‐resolution confocal image stacks. While the microglial density was similar in the wild type and knock‐out mice (Figure 1a,b), their morphology was significantly different. The P2Y_13_ KO microglia displayed a much less ramified morphology (Figure 1c), with shorter and fewer complex processes (Figure 1d), covering less volume than their WT counterparts. Sholl analysis‐derived quantification showed that knocking out P2Y_13_ receptors reduced the number of intersections of microglial processes with concentric Sholl spheres at increasing distances from the soma (Figure 1e), and reduced numbers of process terminals (tips; Figure 1f) and branch points (Figure 1g). Thus, microglia without P2Y_13_ receptors show a less complex morphology, with fewer and shorter processes compared to their wild‐type counterparts.

**Figure 2:**
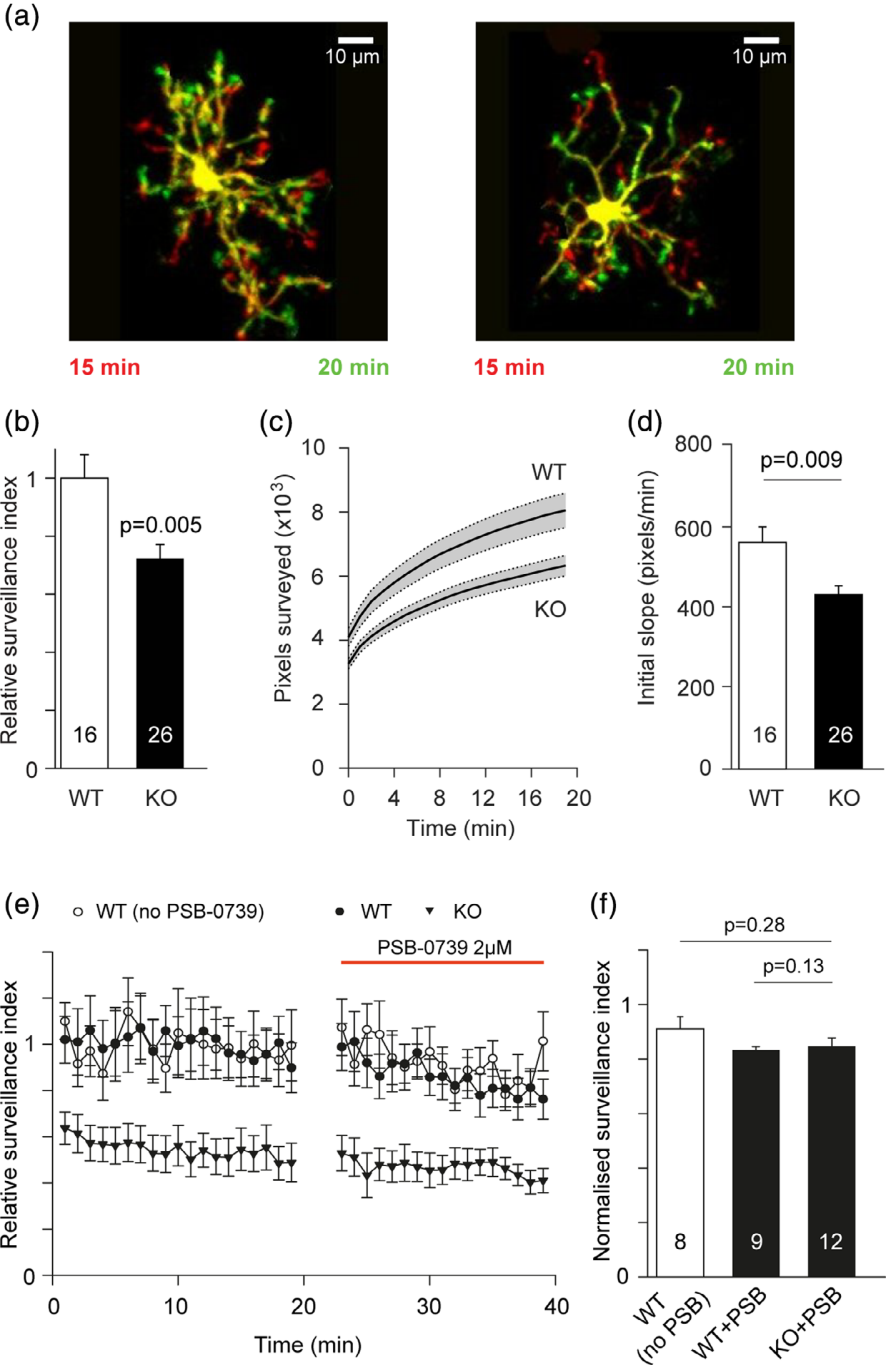
Effect of P2Y_13_ receptor knock‐out on microglial surveillance in hippocampal slices. (a) Specimen images taken 5 min apart of P85 WT and P88 P2Y_13_ KO Iba1‐eGFP microglia, showing process extensions and retractions (red = retracted, green = extended processes) and the less ramified shape of microglia in the KO. (b) Quantification of surveillance for microglia from 3 WT and 3 KO mice, showing less surveillance in the KO than in their WT littermates. (c) Time course of increase of the number of surveyed pixels in maximum intensity projections of images of microglia in WT and KO Iba1‐eGFP mice. Initial value is the area of the cell in the first image frame. (d) Initial slope of graphs in c (measured over the first 2 min, when assessment of surveillance is least confounded by pixel overlap in the maximum intensity projection. (e) Time course of surveillance indices with no drugs (no PSB‐0739) in 3 WT mice or with application after 20 min of PSB‐0739 (PSB) to block P2Y_12_ receptors in 3 WT and 3 KO Iba1‐GFP hippocampal slices. Data show less surveillance in the KO and no significant effect of PSB. Data are normalized to the mean baseline values of the WT mice (averaged over the period from 1 to 19 min). (f) Quantification of the normalized surveillance index in the presence of PSB‐0739, calculated as the mean surveillance index in PSB‐0739 (averaged over the last 5 min in the drug) relative to the mean baseline surveillance index (averaged over the last 5 min of the baseline). The value shown for the Control bar is obtained from data at the same times as for the slices to which PSB‐0739 was applied, but for the WT slices to which no PSB was applied. Age for b–f was P85–P119 for WT and P88–P131 for KO. Number of microglia shown on bars; *p* values were from *t* tests [Color figure can be viewed at http://wileyonlinelibrary.com]

To investigate how microglial surveillance is affected by knocking out P2Y_13_ receptors, we live‐imaged microglia genetically labeled with eGFP in hippocampal slices from WT and P2Y_13_ KO mice using two‐photon microscopy, and quantified changes of surveillance minute‐by‐minute (see section 2). Experiments were carried out less than 4 hr after brain slicing, on microglial cells located 50–100 μm deep in the slice, to avoid microglial activation (Hanisch & Kettenmann, 2007; Kurpius et al., 2006). Briefly, the surveillance index is a measure of the number of image pixels surveyed per unit time and depends on both the number of cell processes and their speed of movement. Knocking out P2Y_13_ receptors significantly reduced surveillance of the brain by microglia (Figure 2a,b). Plotting the time course of the increase in surveyed area in maximum intensity projections showed that the initial rate of surveillance was reduced by 23% (*p* = .009), and the cumulative area (in maximum intensity projections) surveyed after 20 min was reduced by 21% (*p* = .005) in the P2Y_13_ KO mice (Figure 2c,d; Movie S1). This decrease in surveillance may, at least in part, reflect the fact that microglia in P2Y_13_ KO mice are less branched and/or less motile than in WT mice. Analyzing the ramification index—a measure of the ratio of the cell's perimeter to its area (normalized to that of a circle of the same area) which depends on the cell's shape but not on its overall size (Madry, Kyrargyri, et al., 2018)—showed that KO cells had a ramification index that was 11% smaller (*p* = .01) than WT cells. Further analysis of the cell size‐independent motility index—derived by normalizing the surveillance index to the mean area of each cell—showed that the KO cells, despite being less ramified, are as motile as the WT ones (motility index KO: 0.153 ± 0.008 and WT: 0.162 ± 0.007; *p* = .477). Thus, the decreased surveillance of the P2Y_13_ KO microglial cells largely arises from their decreased ramification.

To test whether these effects may originate from elevated cAMP levels due to the disruption of the major P2Y_13_‐gated G_i_‐protein coupled signaling pathway (Bernier et al., 2019), while imaging WT brain slices we applied forskolin, which activates adenylate cyclase and raises cAMP. This reduced surveillance by 43% (*p* = 1.2 × 10^−9^, Figure S2a,c,d) and ramification by 22% (*p* = 3.5 × 10^−11^, Figure S2a,e,f), suggesting that the altered morphology and surveillance of P2Y_13_ KO microglia, which resemble what occurs after forskolin treatment in WT slices, may be mediated by a rise in cAMP levels.

These results show that microglia in mice without P2Y_13_ receptors are less ramified than microglia in WT mice, and survey less brain volume per unit time than their wild‐type counterparts, thus requiring a longer time to survey a given brain volume.

### 3.2 Microglial surveillance still occurs in the absence of P2Y_12_ and P2Y_13_ function

We have recently shown that short‐term block of P2Y_12_ receptors with the selective antagonist PSB‐0739 does not alter surveillance of the brain by microglia, implying that the normal level of ADP in the extracellular space of brain slices is too low to activate P2Y_12_ receptors (Madry, Kyrargyri, et al., 2018). P2Y_13_ receptors have a similar affinity for ADP (Abbracchio et al., 2006), and so are also not expected to be activated by ambient ATP/ADP levels. Nevertheless, to test whether physiological levels of ATP/ADP in the brain can regulate surveillance through P2Y_13_, we first imaged microglial cells in slices of WT and P2Y_13_ KO mice in the presence of MRS 2211 (25 μM), a commonly used antagonist for P2Y_13_ receptors, and measured their surveillance and ramification. We found that MRS 2211 reduced both parameters in wild‐type microglia, but surprisingly also reduced them (by a similar amount) in P2Y_13_ KO microglia (Figure S3a,b). This is inconsistent with the suppression of surveillance being mediated by block of P2Y_13_ receptors. Indeed, MRS 2211, which is a competitive antagonist of P2Y_13_ receptors (pIC_50_ = 5.97, in the presence of 100 nM ADP) is also an antagonist, albeit 20‐fold less powerful (Kim et al., 2005), for P2Y_12_ receptors and for P2Y_1_ receptors (expressed by neurons and astrocytes; Zhang et al., 2014). However, neither activation nor blockade of P2Y_1_ receptors, by applying MRS 2365 (10 μM) and MRS 2179 (25 μM), respectively, altered surveillance (see Figure S4a,c), while MRS 2365 weakly reduced ramification (Figure S4b,d). These data, in combination with the lack of effect on surveillance of the P2Y_12_ receptor blocker PSB‐0739 (Madry, Kyrargyri, et al., 2018), imply that the MRS 2211‐evoked effects do not reflect an action on P2Y_1_ or P2Y_12_ receptors.

We considered the possibility that P2Y_13_ and P2Y_12_ might affect each other's expression, or form dimers and act cooperatively perhaps because each receptor affects the trafficking of the other to the cell surface membrane (see next section) or because of a need for ADP to bind to both P2Y_12_ and P2Y_13_ (assuming a dimeric receptor) for receptor activation to occur. We initially quantified the total P2Y_12_ protein level in the P2Y_13_ KO brain tissue by western blot and found that it was not significantly different compared to the WT tissue (Figure S5a,b). Immunohistochemistry with a P2Y_12_ antibody also showed that its protein distribution in the P2Y_13_ KO microglia in fixed hippocampal slices was broadly similar to that in wild‐type microglia (Figure S5c). To test the effect on microglial surveillance of blocking both P2Y_12_ and P2Y_13_ receptor function, we applied the selective P2Y_12_ antagonist PSB‐0739 to P2Y_13_ KO slices and quantified any change in surveillance index that occurred. Blocking P2Y_12_ receptors with PSB‐0739 (2 μM) did not significantly affect surveillance of microglia in the P2Y_13_ KO or WT (Figure 2e,f). This confirms our previous finding that the physiological levels of ATP and ADP in the brain are too low to affect microglial surveillance by activating P2Y_12_ receptors (Madry, Arancibia‐Cárcamo, et al., 2018; Madry, Kyrargyri, et al., 2018) and presumably also P2Y_13_ receptors, which have a similar high affinity for ADP (Abbracchio et al., 2006).

In summary, knocking out P2Y_13_ receptors reduces microglial ramification and thus surveillance of the brain. These effects were also seen with the supposedly specific P2Y_13_ blocker MRS 2211, but MRS 2211 also had this effect in the P2Y_13_ KO and so acts by a mechanism not involving P2Y_13_ receptors. Surveillance still occurred with P2Y_12_ receptors blocked in the P2Y_13_ knock‐out, implying that no purinergic stimulation of either of these receptors is needed for microglial surveillance to occur.

### 3.3 Microglia lacking P2Y_13_ receptors show a normal resting potential but a larger ADP‐evoked K^+^ current

To test whether the effect of knocking out P2Y_13_ receptors on ramification and surveillance was mediated by changes of the membrane potential, as found previously when blocking THIK‐1 K^+^ channels in the microglial membrane (Madry, Kyrargyri, et al., 2018), we performed whole‐cell patch‐clamp experiments on microglia labeled with fluorescently tagged isolectin B_4_ in situ in acute WT and P2Y_13_ KO mouse hippocampal slices.

**Figure 3:**
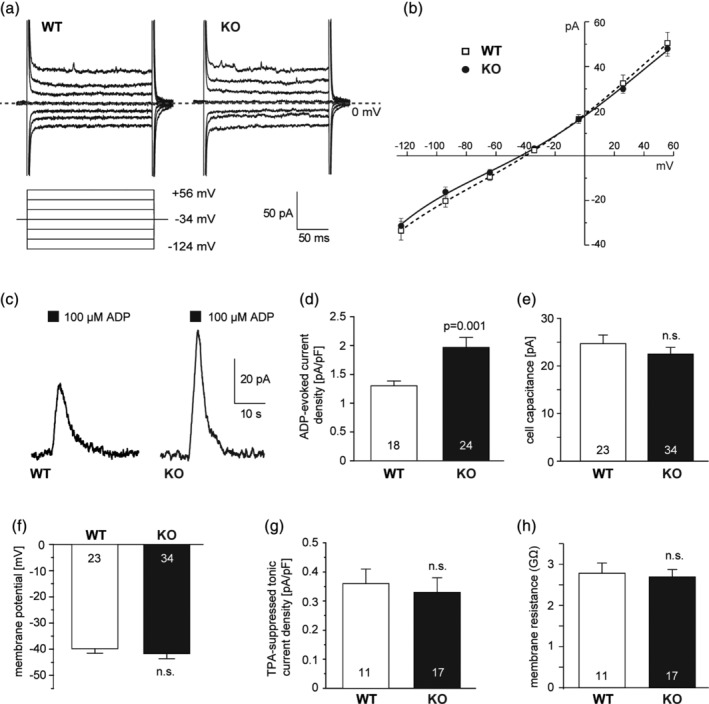
P2Y_13_ knock‐out increases ATP‐evoked membrane current changes but not tonic activity of THIK‐1 K^+^ channels. (a) Specimen current responses of microglia in WT and P2Y_13_ KO hippocampal slices to voltage steps between −124 and +56 mV, from a holding potential of −34 mV. (b) Mean steady‐state *I*–*V* relations from data as in (a) from 10 WT and 12 KO cells. (c) Peak outward current evoked at 0 mV in WT and KO microglia by puffing 100 μM ADP above the slice. (d) Mean ADP‐evoked current density (number of cells on bars). (e–g) Mean cell capacitance (e) resting potential (f), tetrapentylammonium‐suppressed tonic THIK‐1 current density (g), and input resistance (h) in WT and KO cells (numbers of cells on bars). All data from P60‐120 littermate‐matched mice

Both WT and P2Y_13_ KO microglia had a resting potential of ~ −40 mV and showed an approximately linear *I*–*V* relation with slight outward rectification, indicating the absence of voltage‐dependent conductances (Figure 3a,b). Applying ADP from a puff pipette (containing 100 μM ADP) located above the slice evoked an outward current at 0 mV as reported previously (Madry, Kyrargyri, et al., 2018), however in the P2Y_13_ KO the current density was ~50% larger (Figure 3c,d). The ADP‐evoked current reflects the activation of THIK‐1 K^+^ channels by ADP binding to P2Y_12_ receptors but THIK‐1 channels are also tonically active and thus set the resting potential (Madry, Kyrargyri, et al., 2018). Despite the increase of the ADP‐evoked current in the P2Y_13_ KO, the tonic activity of THIK‐1 was unaffected, since the resting potential, the tetrapentylammonium (TPA) sensitive tonic THIK‐1 current component and the input resistance of the cells were all unaffected by KO of P2Y_13_ (as was the cell capacitance: Figure 3e–h, which may reflect an increase in process diameter compensating for shorter process length). This suggests that deleting P2Y_13_ increases the function of P2Y_12_ receptors (as assessed by their coupling to THIK‐1: see section 4). The enhanced P2Y_12_ receptor signaling in the P2Y_13_ KO might suggest that damage‐evoked ATP/ADP release and activation of P2Y_12_ receptors should more effectively attract microglial processes to a damage site (Haynes et al., 2006), but in fact this did not occur as the chemotactic response was attenuated in the KO (see below).

To test whether the larger ADP‐evoked THIK‐1 currents may reflect a raised cAMP level when P2Y_13_ receptors are knocked out (see above), we perfused WT microglial cells for ~10 min with cAMP via the patch solution, which enhanced the ADP‐evoked current density by >70% (*n* = 9; *p* = .017: see Figure S2b). This suggests that the larger ADP‐evoked THIK‐1 currents of P2Y_13_ KO microglia may be mediated via elevated cAMP levels, in line with cAMP mimicking the reduced morphology and surveillance seen in P2Y_13_ KO microglia (Figure S2a,c–f).

### 3.4 P2Y_13_ receptors regulate the rate of microglial directed motility

**Figure 4:**
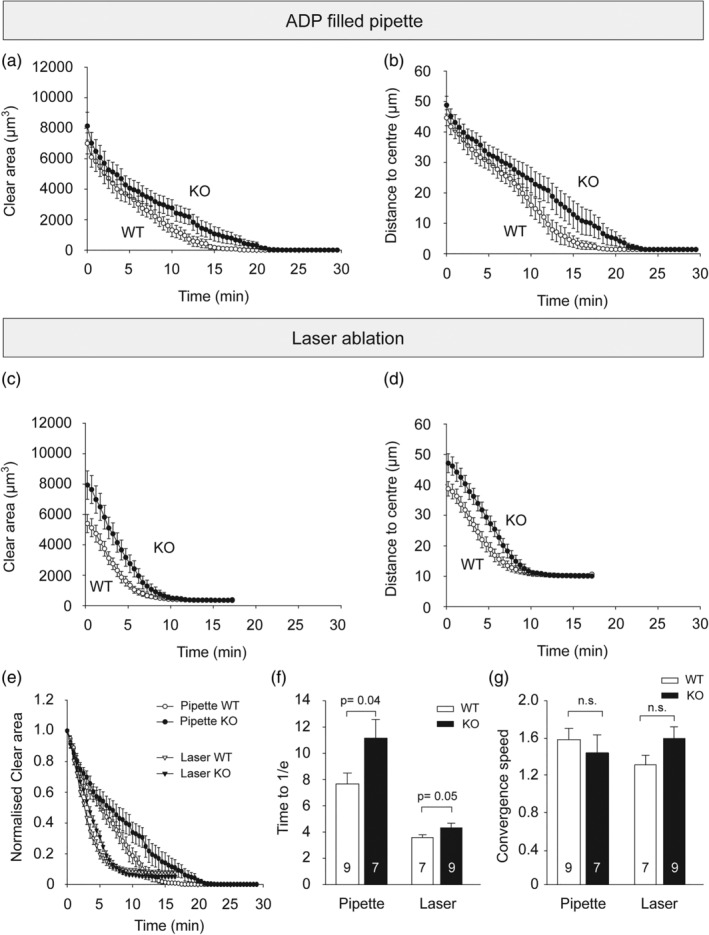
Effect of P2Y_13_ receptor knock‐out on microglial directed motility in hippocampal slices. (a‐b) Directed motility to an ATP‐filled pipette. (a) Time course of directed motility quantified as reduction of the “clear area” not occupied by microglia around the 1 mM ADP‐filled pipette tip in 6 WT and 5 KO mice. (b) Time course of directed motility quantified as reduction of the mean distance (μm, averaged over sectors) from the microglial processes to the ADP‐filled pipette tip in 6 WT and 5 KO mice. (c,d) Directed motility to a laser ablation. (c) Time course of directed motility quantified as reduction of the “clear area” not occupied by microglia around the ablation site in 3 WT and 3 KO mice. (d) Time course of directed motility quantified as reduction of the mean distance (μm, averaged over sectors) from the microglial processes to the ablation site in three WT and three KO mice. (e) Time course of directed motility quantified as reduction of the normalized “clear area” around the ADP‐filled pipette in 6 WT and 5 KO mice and around the laser ablation site in 3 WT and 3 KO mice. The values were normalized to the initial “clear area” values of each recording. (f) Quantification of the time needed for the plots in (e) to drop to the 1/*e* value, that is, the time needed for the “clear area” to drop 36.7% of the initial area. (g) Quantification of the speed of convergence of the WT and KO microglial processes onto the tip of the pipette and the laser ablation site, calculated as the distance (μm) moved per 30 sec, averaged over the period needed to converge on the pipette tip or the ablation site, and then averaged over all the slices. Number of slices are shown on bars. Age for pipette experiments (a, b, e–g) was P55–P121 for WT and P56–P136 for KO and for laser ablation experiments (c, d, e–g) was around P60

To investigate a possible effect of P2Y_13_ receptors on microglial directed motility, we imaged slices with two‐photon microscopy in acute hippocampal slices prelabeled with fluorescently tagged isolectin B_4_ (see section 2). Inserting a 1 mM ADP filled glass pipette (with a tip diameter of ~2–3 μm) carefully into the slices induced directed motility of microglial processes to the tip of the pipette. This resulted in complete process convergence on the tip of the pipette in both WT and P2Y_13_ KO slices (Figure 4a,b), however the time needed for the convergence of P2Y_13_ KO microglial processes (time to 1/*e*) was 46% longer than for their WT counterparts (*p* = .04, Figure 4e,f). We also evoked directed motility in slices of both genotypes in response to focal brain injury by applying a laser ablation of ~10 μm diameter (Figure 4c,d). The two methods initiated similar microglial responses but with different kinetics (Figure 4a–d). The laser ablation induced an overall faster microglial response with the complete convergence of processes occurring ~8 min after the ablation, while a chemotaxis induced by locally elevating ADP through a glass pipette took ~15 min (Movie S2). Microglial processes took 22% longer to arrive at the ablation site when P2Y_13_ was knocked out (*p* = .05, Figure 4e,f). Analysis of the processing speed revealed no differences between the WT and the KO (Figure 4g).

Overall, our results show that P2Y_13_ receptors are necessary to maintain an efficient chemotactic response of microglial processes to CNS injury.

### 3.5 P2Y_13_ receptors reduce the resting level of interleukin 1β release

IL‐1β and TNFα are two major pro‐inflammatory cytokines generated in response to infection and contribute to tissue injury during disease. The production of IL‐1β from innate immune cells such as macrophages and microglia requires the formation of inflammasome complexes to activate caspase 1, which generates IL‐1β from its inactive precursor. Inflammasome assembly is a multi‐stage process, involving priming by a Toll‐Like Receptor agonist such as the bacterial coat component lipopolysaccharide (LPS), followed by a fall of intracellular [K^+^] evoked by an activating signal such as ATP (Munoz‐Planillo et al., 2013). We have previously shown that, in combination with priming by LPS, activation of P2Y_12_ and P2Y_13_ receptors with 2‐MeSADP leads to inflammasome‐mediated production of interleukin‐1β: a process in which P2Y_12_ receptors presumably evoke K^+^ loss through the activation of THIK‐1 K^+^ channels (Madry, Kyrargyri, et al., 2018).

**Figure 5:**
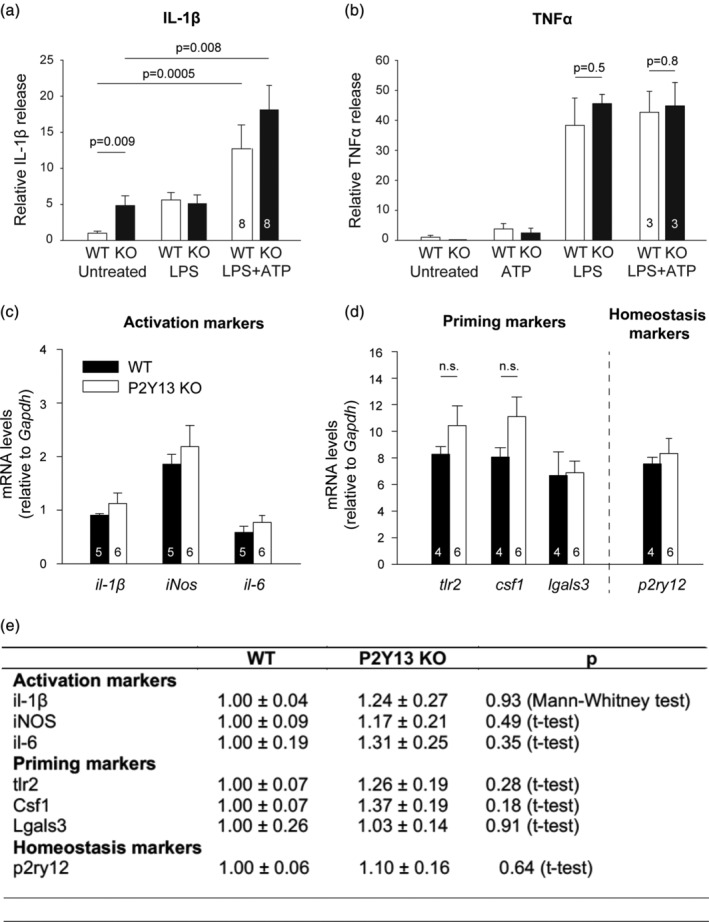
P2Y_13_ KO increases baseline release of interleukin 1β in brain slices, however, the microglia in the intact P2Y_13_ KO brains are not detectably activated. (a) ELISA‐measured IL‐1β levels released from hippocampal slices from 8 WT (at P56–P93) and 8 P2Y_13_ KO mice (at P58–P105) exposed to no drugs, LPS (50 μg/ml), ATP (1 mM), or ATP + LPS, showing an enhanced resting level of IL‐1β release in the KO. (b) ELISA‐measured TNFα levels released from hippocampal slices from 3 WT and 3 P2Y_13_ KO mice exposed to the same protocol as (a), showing a lack of effect of knocking out P2Y_13_ receptors on TNFα release. (c‐d) mRNA levels of selected “activation” (*il6*, *il‐1β*, *iNos*), “priming” (*tlr2*, *csf1*, *lgals3*), and “homeostasis” (*p2ry12)* marker genes in whole brain samples from 5 WT (at P64‐P115, 4 males, 1 female) and P2Y_13_ KO mice (at P62–P115, 4 males, 2 females), relative to Gapdh, as measured by quantitative RT‐PCR. (e) Transcript levels of the selected markers shown in c and d in P2Y_13_ KO brains expressed as *n*‐fold change relative to their WT aged‐matched controls. The number of mice is shown on bars

To determine whether P2Y_13_ receptors play a role in the generation and release of immune mediators when microglia become activated we used acute hippocampal slices from wild type and P2Y_13_ KO mice and quantified (by ELISA) the level of the released IL‐1β in the supernatant (see section 2). Applying LPS (50 μg/ml) for 6 hr evoked some IL‐1β release, which was greatly enhanced when co‐applied with ATP for the last 3 hr of LPS exposure, both in WT and P2Y_13_ KO brain slices (Figure 5a). In the KO, IL‐1β release evoked by LPS and ATP was slightly but not significantly higher than in the WT. However, the level of baseline IL‐1β release in the absence of added LPS or ATP, was significantly higher in P2Y_13_ KO slices than in WT slices (Figure 5a), suggesting that P2Y_13_ receptors suppress the release of IL‐1β in baseline conditions. This may also suggest that microglia exhibit a different functional phenotype under resting conditions, however, P2Y_13_ KO microglia did not appear to be activated in the intact brain as judged by their cell body area (Davis, Salinas‐Navarro, Cordeiro, Moons, & De Groef, 2017; Gyoneva et al., 2014), which was 50.6 ± 3.78 μm^2^ in the WT and 47.0 ± 1.7 μm^2^ in the KO.

We further tested whether the release of another cytokine, TNFα, is affected when P2Y_13_ receptors are deleted using the same procedure as for IL‐1β. TNFα is a proinflammatory cytokine, which is released by glial cells in the brain (mainly microglia and astrocytes) in response to pathogen‐ or damage‐associated molecular patterns, in a process that does not require inflammasome assembly (and so presumably does not require K^+^ loss from the cell via THIK‐1). LPS evoked a similar release of TNFα in WT and P2Y_13_ KO brain slices, which was independent of the presence of ATP (Figure 5b), indicating that P2Y_13_ receptors do not regulate the production of TNFα by microglia.

To further investigate the impact of P2Y_13_ receptor deletion on the functional state of microglia in the intact brain, we evaluated the mRNA expression of selected microglial target genes in whole brains from P2Y_13_ KO mice and aged‐matched WT controls by RT‐PCR. Target genes were selected according to their expression level in different functional states of microglia and were classified as being associated with “activation” (*il1b*, *il6*, *iNos*), “priming” (*tlr2*, *lgals3*, *tlr2*), or “homeostasis” (*p2ry12*), following Holtman et al. (2015). The mRNA expression of all the genes tested by this method was not different in P2Y_13_ KO mice (Figure 5c–e). Thus, our results show that P2Y_13_ receptors suppress the release of IL‐1β in brain slices, however, microglial cells lacking P2Y_13_ receptors were not detectably activated.

## 4 DISCUSSION

P2Y_12_ and P2Y_13_ receptors are both highly expressed in microglia at the mRNA level (Zhang et al., 2014), both use ADP as their preferred physiological agonist with an EC_50_ value of ~60 nM (see overview in Abbracchio et al., 2006) and share a similar pharmacological profile (Zhang et al., 2002). Both receptors couple predominantly to Gα_i_ proteins and so their activation will inhibit the adenylate cyclase and thus decrease intracellular cAMP levels. P2Y_12_ activation has been shown to mediate directed motility of microglial processes to an ATP/ADP source (Haynes et al., 2006), but the function of microglial P2Y_13_ receptors is unknown.

We took advantage of the existence of a P2Y_13_ receptor knock‐out mouse (Fabre et al., 2010) to investigate the role of this receptor in microglial function. Although P2Y_13_ is highly expressed at the mRNA level in microglia (Stefani et al., 2018; Zhang et al., 2014), its function has been little studied. This partly reflects a lack of specific pharmacological tools to activate or inhibit this receptor. MRS 2211 is often used as a “specific” P2Y_13_ receptor blocker, but we found that it affected microglia even when P2Y_13_ receptors were knocked out, thus implying that it is not a specific tool to study the role of P2Y_13_ receptors in any tissue. Haynes et al. (2006) and Stefani et al. (2018) found that, despite confirming P2Y_13_ mRNA expression in microglia (Zhang et al., 2014), its expression could not be detected at the protein level. The inability to antibody‐label P2Y_13_ may reflect a high rate of constitutive proteasomal degradation (Pons et al., 2014), or may alternatively suggest that P2Y_13_ protein is only expressed at high levels in microglial culture (Quintas, Vale, Goncalves, & Queiroz, 2018) or upon microglial activation in situ (Niu et al., 2017).

We found that knock‐out of P2Y_13_ receptors had four main effects on microglia. (a) It reduced their ramification (Figure 1) which consequently reduced their surveillance of the brain parenchyma. (b) It enhanced the outward THIK‐1 K^+^ current evoked by ADP activating P2Y_12_ receptors. This is mediated by an increased coupling of P2Y_12_ receptors to THIK‐1 channels (and not an increased number of THIK‐1 channels) since the tonic activity of THIK‐1 and thus the resting potential was not changed (Figure 3). (c) Surprisingly, despite the potentiation of P2Y_12_ receptor function as assessed by ADP‐evoked THIK‐1 activation, the time needed for P2Y_12_ receptor‐mediated convergence of microglial processes to an ADP source or to local injury triggered by a laser ablation was prolonged in the P2Y_13_ KO (Figure 4). (d) The release of the pro‐inflammatory cytokine IL‐1β was increased in the P2Y_13_ KO in the absence of stimulation with LPS and ATP (Figure 5), suggesting the adoption of a different functional state with some resemblance to mild activation, although microglial cells were not detectably activated as judged by the lack of expression of classical microglial activation markers (Figure 5). Thus, P2Y_13_ contributes to maintaining microglial morphology and surveillance and suppresses inappropriate IL‐1β release.

The attraction of microglial processes to an ATP source or laser lesion involves activation of P2Y_12_ receptors by ADP (Haynes et al., 2006) and is independent of THIK‐1 activity (Madry, Kyrargyri, et al., 2018). P2Y_12_ receptor protein levels were not changed in the P2Y_13_ KO (at least at the level of the whole brain: Figure S5) and the P2Y_13_ KO microglia show an enhanced THIK‐1 K^+^ current (Figure 3) evoked by ADP (Madry, Kyrargyri, et al., 2018), which suggests that the sensitivity of the P2Y_13_ KO cells to an ADP source is increased. This increase may reflect more trafficking to the membrane of P2Y_12_ receptors in the P2Y_13_ KO, better coupling of P2Y_12_ to THIK‐1 in the absence of P2Y_13_, or alternatively an effect of the increase of intracellular cyclic AMP level (e.g., altering phosphorylation and potentiating P2Y_12_ action) expected when the G_i_‐coupled P2Y_13_ is knocked out (assuming that there is constitutive activity of P2Y_13_, which is not known). This is in line with our data showing that an elevation of cAMP levels, either by applying forskolin to WT slices or perfusing cAMP intracellularly via the patch‐clamp solution, causes WT microglial cells to adopt features seen in P2Y_13_ KO microglia, such as reduced surveillance and ramification (consistent with Bernier et al., 2019), and a larger P2Y_12_‐activated THIK‐1 current (Figure S2b). Despite this increased sensitivity to ADP, the P2Y_13_ KO cells moved their processes at a similar speed to those of the WT towards an ADP source, where they nevertheless arrived later because the KO cells are less ramified and have shorter processes, and thus they have to grow further than the WT cell processes until they reach their target.

Previously we have shown that, in nonactivated microglia, ramification is controlled by the cell's membrane potential, so that microglia become less ramified when the cell is depolarized (Madry, Arancibia‐Cárcamo, et al., 2018; Madry, Kyrargyri, et al., 2018). The loss of ramification seen upon deleting P2Y_13_ cannot be explained in this way because the resting potential of the cells was unchanged (Figure 3e), and may arise from the fact that the microglia have adopted a different functional state (Norden & Godbout, 2013; Perry & Holmes, 2014). RT‐PCR analysis of the two genotypes at the whole‐brain level did not reveal differences of the activation state of P2Y_13_ KO microglia (Figure 5c,e), but this may reflect a lack of sensitivity of the assay. The fact that more IL‐1β protein is released in the P2Y_13_ KO (Figure 5a), while the mRNA level for *il‐1β* is unchanged (Figure 5c,e) may reflect a poor correlation between changes of protein level and transcriptional, translational, and post‐translational processes, which has previously been described across different systems, conditions, and developmental stages (Liu, Beyer, & Aebersold, 2016; Peshkin et al., 2015; Taniguchi et al., 2010).

We suggest above that some of the effects of P2Y_13_ deletion may reflect an increase of cAMP concentration in the cell, but other signaling pathways may also be associated with the P2Y_13_ receptor. These include G_q_‐coupled elevations of intracellular [Ca^2+^] mediated by internal store release and activation of store‐operated calcium channels (Carrasquero et al., 2009; Zeng et al., 2014), Erk1/2 activation (Ortega, Perez‐Sen, Delicado, & Teresa Miras‐Portugal, 2011), and regulation of transcription through a rise in nuclear calcium level and activation of nuclear factor erythroid‐derived 2‐like 2, a transcription factor that regulates the oxidative stress response (Espada et al., 2010; Lyubchenko et al., 2011). P2Y_13_ may also inhibit Akt (Chatterjee & Sparks, 2012), which is an important mediator of P2Y_12_‐mediated actin re‐organization. In addition, the levels of P2Y_13_ may directly influence the levels of other purinergic receptors found at the microglial membrane due to the influence that dimerization between different P1/P2 receptors can have on receptor trafficking and retention at the cell surface (Schicker et al., 2009). However, much of the evidence pertaining to the downstream signaling pathways associated with P2Y_13_ should be interpreted with caution, especially in microglia, for the following reasons. First, many studies were conducted in cell types other than microglia, or in cell lines or cultured cells, and there may be differences in the signaling between cell types. Second, almost all of these studies relied highly upon the use of MRS 2211 to block P2Y_13_ receptors, but we have now shown this is not a selective P2Y_13_ antagonist. Furthermore, the failure, to date, to demonstrate microglial P2Y_13_ protein expression in vivo suggests either that the commercially available P2Y_13_ antibodies lack specificity or that the constitutive proteasomal degradation of the receptor shown to regulate the surface expression of the receptor in hepatocytes (Pons et al., 2014) also happens in microglia in vivo (which would imply that a low level of surface receptors can still have a significant effect on cell function).

The high mRNA expression level for P2Y_13_ in microglia, and the P2Y_13_ KO‐evoked effects on microglial function that we have characterized, both suggest that P2Y_13_ plays a direct role in regulating microglial function. Our finding that knocking out P2Y_13_ receptors results in less ramified microglia in the brain under resting conditions is in line with recent studies showing a general reduction in the structural complexity of microglia in P2Y_13_ KO mice (Stefani et al., 2018) and in mice with constitutive deletion of the ATP degrading enzymes CD39 and CD73 (which prevents hydrolysis of extracellular ATP and thus the formation of the P2Y_13_ receptor agonist ADP; Matyash et al., 2017). Nevertheless, the P2Y_13_ knock‐out mouse that has been used is a global KO, and so the effects seen could in principle be mediated by a loss of P2Y_13_ elsewhere in the body. Expression of P2Y_13_ receptor mRNA occurs not only in the brain, but also in the liver, spleen, lymph nodes, and bone (Communi et al., 2001). P2Y_13_ KO mice show a decrease in hepatic high‐density lipoprotein (HDL) uptake, hepatic cholesterol content, and biliary cholesterol output, although their plasma HDL and other lipid levels are normal (Fabre et al., 2010). These metabolic changes result in a substantial decrease in the rate of macrophage‐to‐feces reverse cholesterol transport (RCT), a process whereby excess peripheral cholesterol, especially that in macrophage foam cells, is taken up to be incorporated into HDL particles and delivered to the liver for excretion in the feces. Thus, although it has not yet been studied, a rise in microglial cholesterol level might occur as a result of these peripheral changes. Since cholesterol levels regulate microglial function (Bohlen et al., 2017) and high cholesterol is reported to lead to microglial activation (Zatta, Zambenedetti, Stella, & Licastro, 2002), it is conceivable that the effects we report are mediated by altered systemic cholesterol trafficking. Construction of a microglial‐specific P2Y_13_ receptor KO mouse will help further investigations of the role of P2Y_13_ receptors in microglia, without interference from the actions of this receptor in peripheral tissues.

## Supporting information


**Figure S1** Lack of specific P2Y_13_ protein labeling in situ and in vitro using three different antibodies. (a) Confocal images of WT (upper panel) and P2Y_13_ KO (lower panel) fixed hippocampal slices (CA2 area) labeled with chicken anti‐Iba1 antibody (green), rabbit anti‐P2Y_13_ antibody (red, APR017, Alomone labs), and DAPI (blue). The fact that the red signal is seen in both WT and P2Y_13_ KO slices implies that this antibody is not specific for P2Y_13_ in situ. (b) Confocal images of microglial cells acutely isolated from 3 adult WT (upper panel) and 3 P2Y_13_ KO (lower panel) mice and labeled with goat anti‐Iba1 (green) and rabbit anti‐P2Y_13_ (red, Alomone APR017) antibodies. The red signal was similar for the WT and P2Y_13_ KO cells, implying no specificity of this P2Y_13_ antibody in vitro. (c) Western blot showing P2Y_13_ protein expression (the antibody used was the anti‐P2Y_13_ from Abcam, ab108444) in total spleen samples (20 μg/lane) isolated from a WT and a KO mouse, and in brain protein samples (20 μg/lane) isolated from 2 independent WT and 2 independent P2Y_13_ KO mice. The multiple western blot bands, in both WT and KO tissues, indicate no specificity of the P2Y_13_ antibody. The predicted molecular weight of the P2Y_13_ protein was 37–41 kDa, corresponding to the area indicated in red. (d) DAB immunostaining for P2Y_13_ (the anti‐P2Y_13_ was kindly provided by Prof David Julius, UCSF) in representative paraffin section (5 μm) from hippocampus of a WT mouse. Some positive signal (brown) is indicated in the white box. (e) Paraffin sections from the hippocampus of WT (upper panel) and P2Y_13_ (lower panel) KO mice labeled with anti‐P2Y_13_ antibody (brown, Julius lab) and counterstained with hematoxylin (blue nuclei). P2Y_13_ positive cells exist in both WT and KO sections (asterisks), implying no specificity of the positive signal shown in d. (f) Confocal fluorescent images from paraffin hippocampal slices (5 μm) showing no detectable immunoreactivity when stained with anti‐P2Y_13_ (Julius lab), consistent with the results of Haynes et al. (2006).

**Figure S2** Elevation of cAMP increases ADP‐evoked currents and reduces surveillance and ramification. (a) Specimen images taken 5 min apart of a ramified GFP expressing WT microglia, showing process extensions and retractions (red = retracted, green = extended processes) and the less ramified shape when exposed to 25 μM forskolin to raise intracellular cAMP levels. (b) Mean 100 μM ADP‐evoked current densities of WT microglial cells without and with intracellular perfusion of 2 mM cAMP for ~10 min via the patch pipette solution, measured at a holding potential of 0 mV (number of cells on bars). Time courses of surveillance (c) and ramification (e) indices for application of 25 μM forskolin in hippocampal slices with GFP‐labeled WT microglia. Data showing surveillance are normalized to the mean baseline values of the 10 min control period. (d) Quantification of the normalized surveillance index in the presence of 25 μM forskolin, calculated as the mean surveillance index in forskolin (averaged over the last 5 min in the drug) relative to the mean baseline surveillance index (averaged over the last 5 min of the control period). (f) Quantification of cell ramification as in (d) but without normalization of the data. Number of microglia shown on bars; *p* values were from paired *t* tests.

**Figure S3** Effect of MRS 2211 on surveillance and ramification of WT and P2Y_13_ KO microglia. (a) Effect of MRS 2211 (25 μM) on the surveillance index (normalized to its value averaged over the initial 14 min) in 5 and 8 hippocampal slices from 3 WT and 3 P2Y_13_ KO Iba1‐GFP mice, respectively. (b) Effect of MRS 2211 (25 μM) on the microglial ramification index in 5 and 8 hippocampal slices from 3 WT and 3 P2Y_13_‐KO Iba1‐GFP mice. Age for a and b was P85–P93 for WT and P82–P105 for KO.

**Figure S4** Lack of effect of P2Y1 receptor signaling on microglial surveillance. Time courses of surveillance and ramification indices for application of 25 μM of the P2Y1 receptor antagonist MRS 2179 (a, b; *n* = 17) and for application of 10 μM of the P2Y1 receptor agonist MRS 2365 (c, d; *n* = 11) in hippocampal slices with GFP‐labeled WT microglia. Data showing surveillance are normalized to the mean baseline values of the 10 min control period. *p* values were from paired *t* tests, averaged over the last 5 min of control and drug exposure, respectively. n.s. indicates *p* > .05.

**Figure S5** P2Y_12_ protein expression is unaltered in P2Y_13_ KO brains. (a) Western blot showing P2Y_12_ protein expression in total brain protein samples (20 μg/lane) isolated from 4 independent WT (at P47–P65) and 4 independent P2Y_13_ KO (at P46–P48) mice. Actin is shown as a loading control. (b) Quantification of the western blot shown in (a). (c) Confocal images of WT (P51, upper panel) and P2Y_13_ KO (P48, lower panel) hippocampal slices (CA2 area) immune‐labeled with anti‐P2Y_12_ (green) and DAPI (blue) confirm no obvious change of P2Y_12_ expression in the P2Y_13_ KO.

**Movie S1** P2Y_13_ KO decreases surveillance by microglial processes. Surveillance of the CNS by microglia in hippocampal slices from wild‐type (P85) and P2Y_13_ knock‐out (P88) mice, shown as maximum intensity projections. Left panel of the triptych: process movements of eGFP labeled microglial cell. Middle panel: binarized version of left panel. Right panel: cumulative plot, with each pixel remaining white after a microglial process has entered it, showing how the cell gradually scans the brain volume.

**Movie S2** Different kinetics of microglial directed motility to a patch pipette filled with ADP and to a laser ablation. Directed motility of microglial processes in WT hippocampal slices toward (left) a pipette filled with 1 mM ADP in external solution and (right) to a laser ablation. The microglia are labeled with isolectin B4‐Alexa 594, which also labels blood vessels (large stable structures on the images). Scale bar 30 μm.

## Data Availability

Data available on request from the authors.
