# Supplementary figures and images for: P2Y13 receptors regulate microglial morphology, surveillance, and resting levels of interleukin 1β release
Source: Glia. 2019 Sep 14;68(2):328–44. doi: 10.1002/glia.23719 (PMC6916289; doi:10.1002/glia.23719)

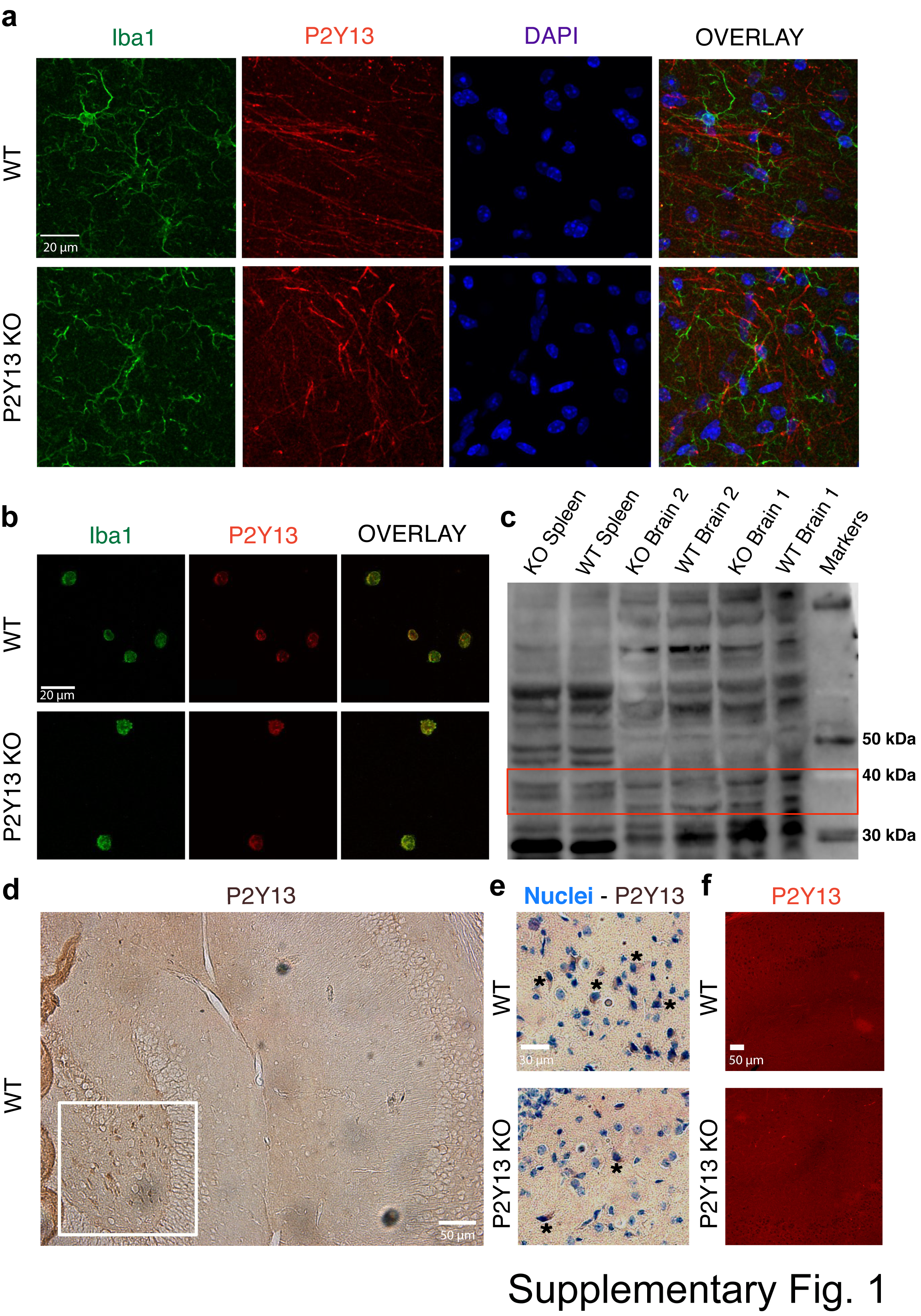

Supplement: Supplementary file 1 — Figure S1 Lack of specific P2Y13 protein labeling in situ and in vitro using three different antibodies. (a) Confocal images of WT (upper panel) and P2Y13 KO (lower panel) fixed hippocampal slices (CA2 area) labeled with chicken anti‐Iba1 antibody (green), rabbit anti‐P2Y13 antibody (red, APR017, Alomone labs), and DAPI (blue). The fact that the red signal is seen in both WT and P2Y13 KO slices implies that this antibody is not specific for P2Y13 in situ. (b) Confocal images of microglial cells acutely isolated from 3 adult WT (upper panel) and 3 P2Y13 KO (lower panel) mice and labeled with goat anti‐Iba1 (green) and rabbit anti‐P2Y13 (red, Alomone APR017) antibodies. The red signal was similar for the WT and P2Y13 KO cells, implying no specificity of this P2Y13 antibody in vitro. (c) Western blot showing P2Y13 protein expression (the antibody used was the anti‐P2Y13 from Abcam, ab108444) in total spleen samples (20 μg/lane) isolated from a WT and a KO mouse, and in brain protein samples (20 μg/lane) isolated from 2 independent WT and 2 independent P2Y13 KO mice. The multiple western blot bands, in both WT and KO tissues, indicate no specificity of the P2Y13 antibody. The predicted molecular weight of the P2Y13 protein was 37–41 kDa, corresponding to the area indicated in red. (d) DAB immunostaining for P2Y13 (the anti‐P2Y13 was kindly provided by Prof David Julius, UCSF) in representative paraffin section (5 μm) from hippocampus of a WT mouse. Some positive signal (brown) is indicated in the white box. (e) Paraffin sections from the hippocampus of WT (upper panel) and P2Y13 (lower panel) KO mice labeled with anti‐P2Y13 antibody (brown, Julius lab) and counterstained with hematoxylin (blue nuclei). P2Y13 positive cells exist in both WT and KO sections (asterisks), implying no specificity of the positive signal shown in d. (f) Confocal fluorescent images from paraffin hippocampal slices (5 μm) showing no detectable immunoreactivity when stained with anti‐P2Y13 (Juliu [file GLIA-68-328-s001.tif]

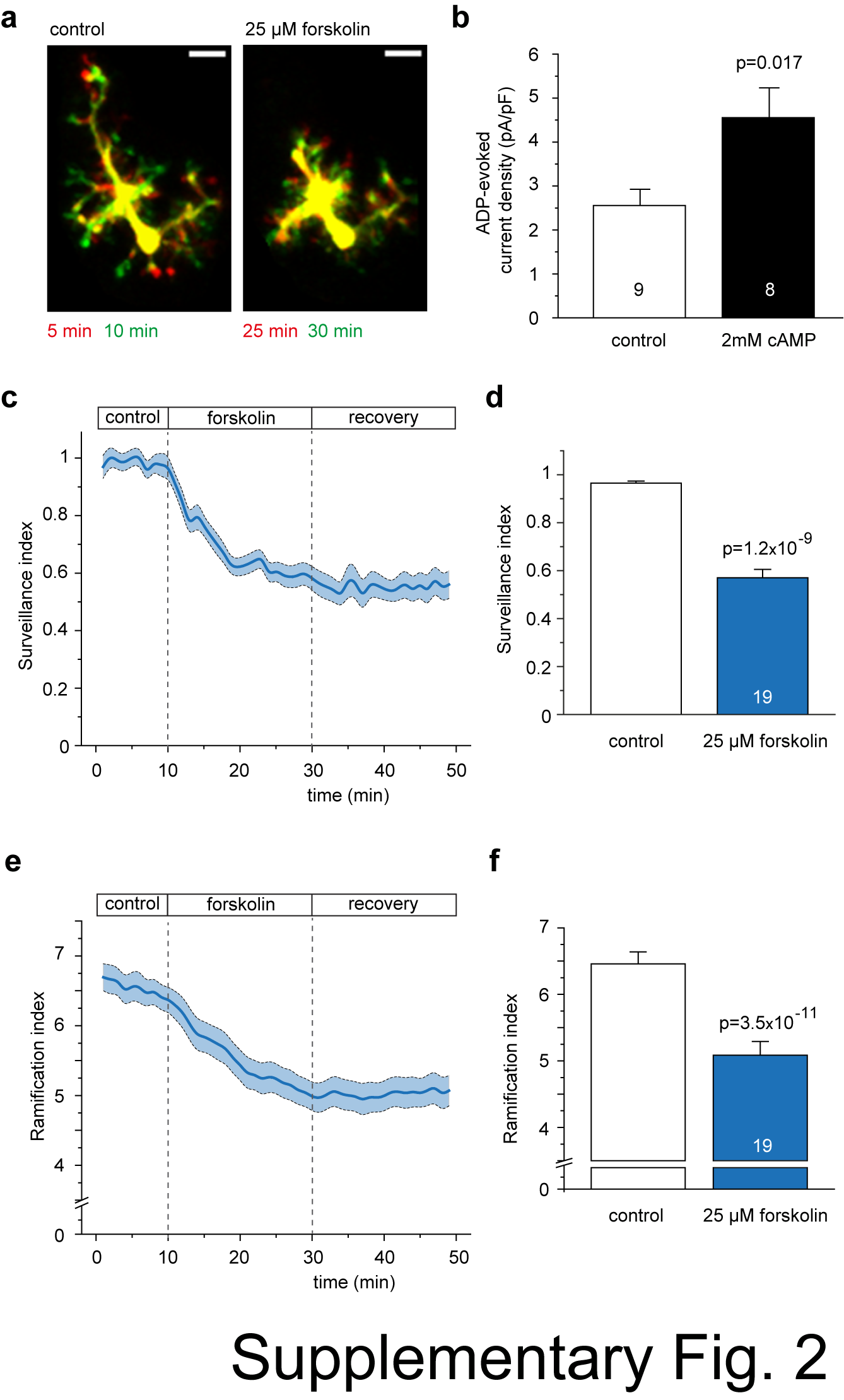

Supplement: Supplementary file 2 — Figure S2 Elevation of cAMP increases ADP‐evoked currents and reduces surveillance and ramification. (a) Specimen images taken 5 min apart of a ramified GFP expressing WT microglia, showing process extensions and retractions (red = retracted, green = extended processes) and the less ramified shape when exposed to 25 μM forskolin to raise intracellular cAMP levels. (b) Mean 100 μM ADP‐evoked current densities of WT microglial cells without and with intracellular perfusion of 2 mM cAMP for ~10 min via the patch pipette solution, measured at a holding potential of 0 mV (number of cells on bars). Time courses of surveillance (c) and ramification (e) indices for application of 25 μM forskolin in hippocampal slices with GFP‐labeled WT microglia. Data showing surveillance are normalized to the mean baseline values of the 10 min control period. (d) Quantification of the normalized surveillance index in the presence of 25 μM forskolin, calculated as the mean surveillance index in forskolin (averaged over the last 5 min in the drug) relative to the mean baseline surveillance index (averaged over the last 5 min of the control period). (f) Quantification of cell ramification as in (d) but without normalization of the data. Number of microglia shown on bars; p values were from paired t tests. [file GLIA-68-328-s002.tif]

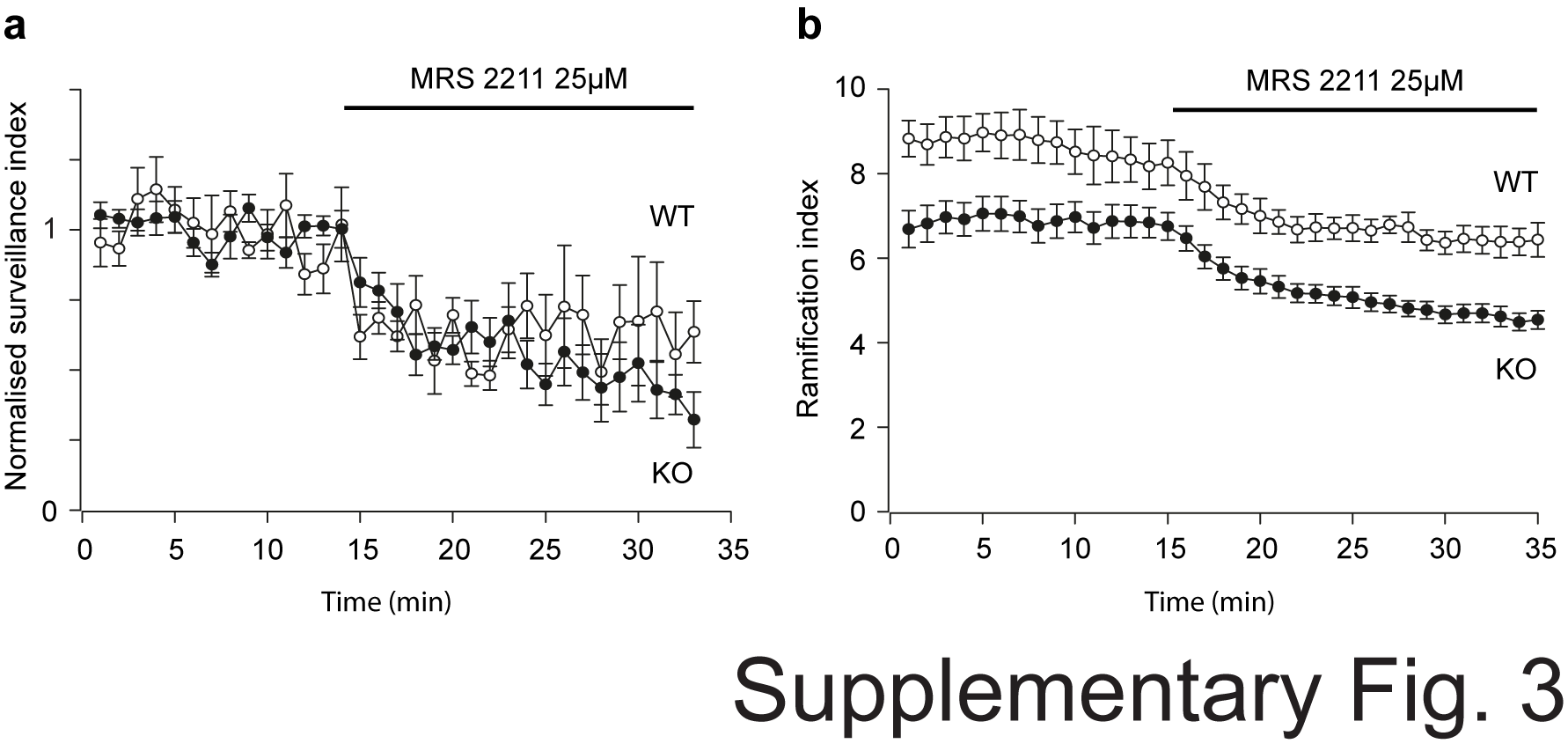

Supplement: Supplementary file 3 — Figure S3 Effect of MRS 2211 on surveillance and ramification of WT and P2Y13 KO microglia. (a) Effect of MRS 2211 (25 μM) on the surveillance index (normalized to its value averaged over the initial 14 min) in 5 and 8 hippocampal slices from 3 WT and 3 P2Y13 KO Iba1‐GFP mice, respectively. (b) Effect of MRS 2211 (25 μM) on the microglial ramification index in 5 and 8 hippocampal slices from 3 WT and 3 P2Y13‐KO Iba1‐GFP mice. Age for a and b was P85–P93 for WT and P82–P105 for KO. [file GLIA-68-328-s003.tif]

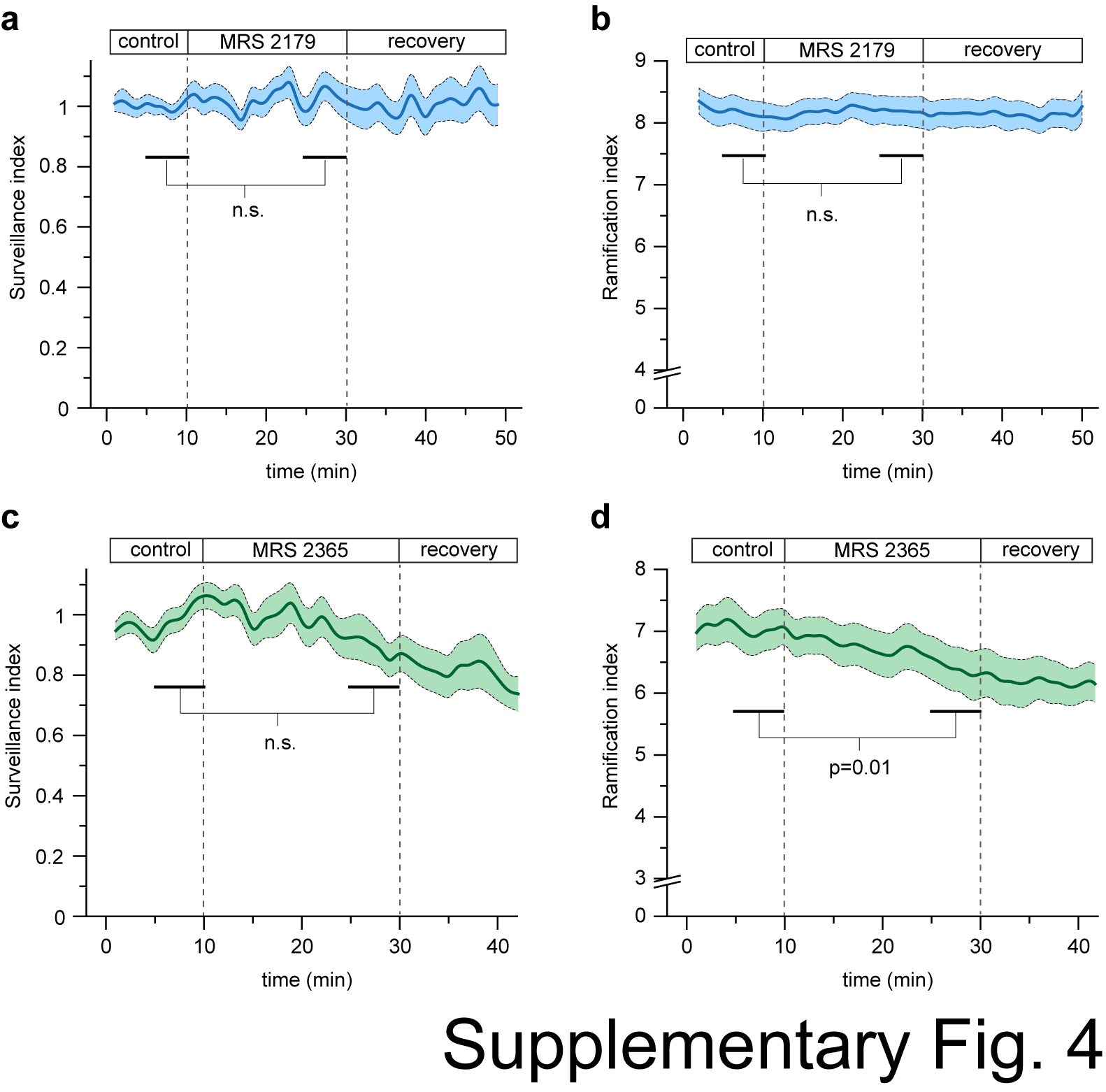

Supplement: Supplementary file 4 — Figure S4 Lack of effect of P2Y1 receptor signaling on microglial surveillance. Time courses of surveillance and ramification indices for application of 25 μM of the P2Y1 receptor antagonist MRS 2179 (a, b; n = 17) and for application of 10 μM of the P2Y1 receptor agonist MRS 2365 (c, d; n = 11) in hippocampal slices with GFP‐labeled WT microglia. Data showing surveillance are normalized to the mean baseline values of the 10 min control period. p values were from paired t tests, averaged over the last 5 min of control and drug exposure, respectively. n.s. indicates p > .05. [file GLIA-68-328-s004.tif]

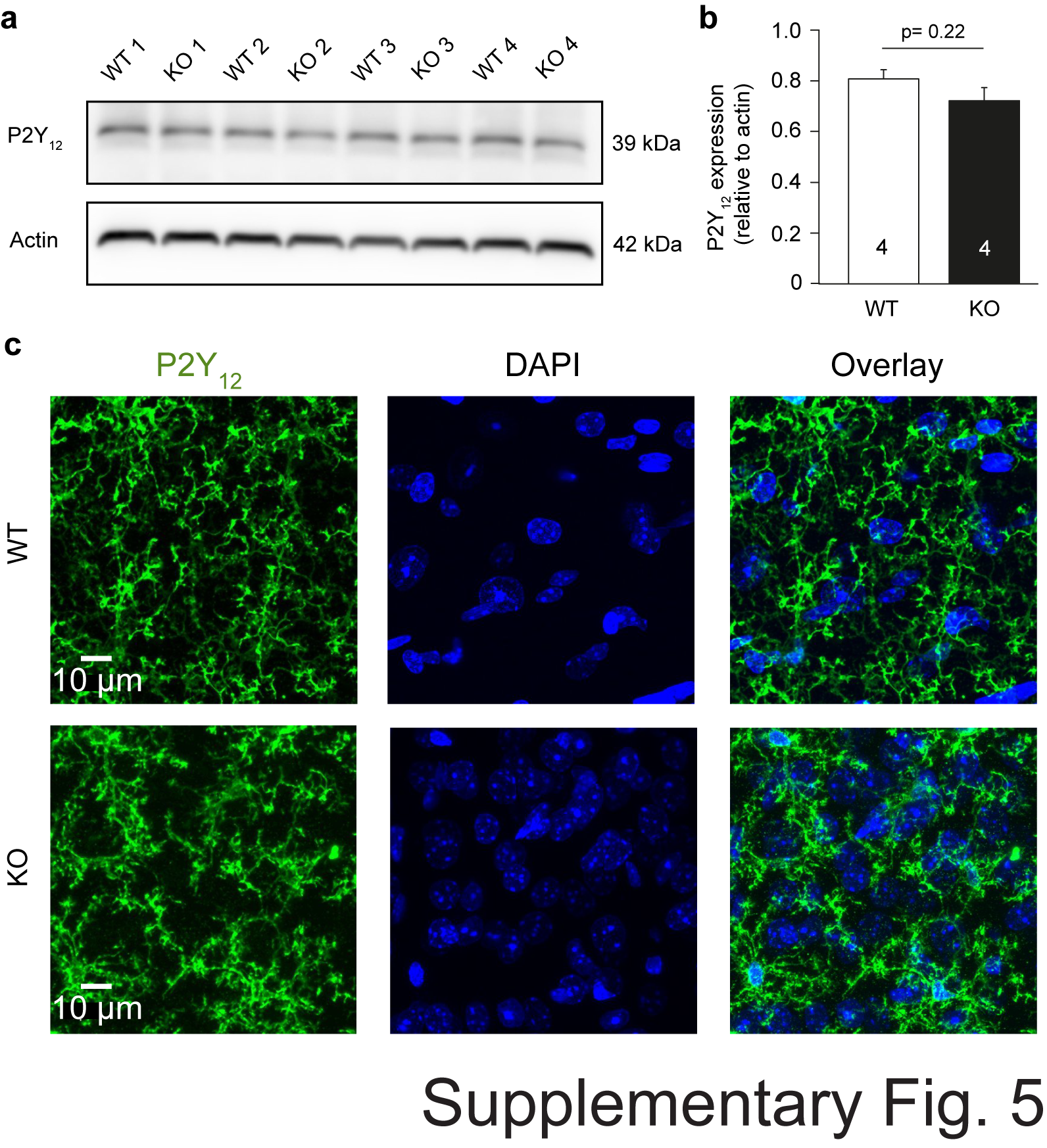

Supplement: Supplementary file 5 — Figure S5 P2Y12 protein expression is unaltered in P2Y13 KO brains. (a) Western blot showing P2Y12 protein expression in total brain protein samples (20 μg/lane) isolated from 4 independent WT (at P47–P65) and 4 independent P2Y13 KO (at P46–P48) mice. Actin is shown as a loading control. (b) Quantification of the western blot shown in (a). (c) Confocal images of WT (P51, upper panel) and P2Y13 KO (P48, lower panel) hippocampal slices (CA2 area) immune‐labeled with anti‐P2Y12 (green) and DAPI (blue) confirm no obvious change of P2Y12 expression in the P2Y13 KO. [file GLIA-68-328-s005.tif]
